# Subchronic and Chronic Toxicity Assessment of Sublancin in Sprague–Dawley Rats

**DOI:** 10.3390/toxics13050413

**Published:** 2025-05-21

**Authors:** Yong Guo, Zhihao Li, Penglong Xu, Gantong Guo, Tao He, Yujiao Lai

**Affiliations:** 1 School of Chemistry and Environmental Engineering, Wuhan Institute of Technology, Wuhan 420200, China; 11809010003@stu.wit.edu.cn; 2 Sinagri YingTai Bio-peptide Co., Ltd., Linzhou 456550, Chinaznytht@126.com (T.H.); 3 Key Laboratory of Feed Antibiotics Replacement Technology, Ministry of Agriculture and Rural Affairs, Linzhou 456550, China; 4 Hubei Key Laboratory of Animal Nutrition and Feed Science, Engineering Research Center of Feed Protein Resources on Agricultural By-Products, Ministry of Education, Wuhan Polytechnic University, Wuhan 430023, China; zhhhli623@163.com; 5 Beijing Key Laboratory of Detection Technology for Animal-Derived Food Safety, College of Veterinary Medicine, China Agricultural University, Beijing 100193, China

**Keywords:** sublancin, SD rat, subchronic toxicity, chronic toxicity, novel veterinary drugs, safety evaluation

## Abstract

Sublancin, an S-linked antimicrobial (glycol) peptide produced by *Bacillus subtilis*, has emerged as a novel and promising veterinary drug due to its unique antibacterial mechanism, low risk of resistance, and properties that modulate the immune system, reduce inflammation, and promote gut health. This study comprehensively assessed the subchronic (90-day) and chronic (180-day) toxicity of Sprague–Dawley (SD) rats, following the guidelines issued by the Ministry of Agriculture of China. Rats were orally administered sublancin at doses of 2000, 10,000, or 50,000 mg/kg feed, representing 1666–5000 times the efficacious dose (1.0–1.2 mg/kg) reported in mice via the same administration route. Throughout this study, a wide range of physiological and behavioral parameters were monitored to access the toxicity of sublancin, including appetite, water intake, body weight gain, and organ weights. Hematological and biochemical analyses, as well as histopathological examinations of the major organs, were conducted at the end of each study period. The results indicated no adverse effects on any measured parameters at any dose level, with no significant differences observed between the sublancin-treated groups and the control group (*p* > 0.05). Notably, even the highest dose of 50,000 mg/kg did not induce growth inhibition or physiological dysfunction. A histopathological examination also revealed no tissue abnormalities in the major organs. The no-observed-effect level (NOEL) was determined to be 50,000 mg/kg for both study periods. These results demonstrate the long-term safety of sublancin in Sprague–Dawley rats, with no adverse effects during 180 days of oral administration at doses 1666–5000-fold the documented antimicrobially effective and immune-enhancing doses.

## 1. Introduction

The rapid growth of modern animal husbandry has led to larger-scale and more intensive livestock and poultry farming, resulting in extensive antibiotic use in animal production. However, misusing and irregularly using antibiotics have increased bacterial resistance and drug residue issues, affecting animal health and meat quality, while also posing potential threats to human health [1]. To tackle these challenges, the Chinese government has banned antibiotics in feed and reduced their use in veterinary drugs. The goal is to reduce antibiotic use, minimize the risk of resistance, and ensure the safety of animal products. Thus, developing novel, safe, and efficient veterinary drugs, especially those with antimicrobial properties that leave no residues, has become a critical research priority in animal husbandry and food safety.

Sublancin, a 37-amino acid antimicrobial (glycol) peptide produced by *Bacillus subtilis 168*, featured two α-helices (Helix A and Helix B) connected by a loop and stabilized by two nested disulfide bonds between four cysteine residues, forming its characteristic helix–loop–helix hairpin structure [2,3]. This S-linked (glycol) peptide contains a crucial glucose modification at Cys22, with both glycosylation and the disulfide-bonded structure being essential for its antimicrobial activity [3]. Functionally, sublancin exhibits broad-spectrum efficacy against Gram-positive bacteria in vitro at a minimum inhibitory concentration (MIC) of 15 μM [4] (e.g., *Staphylococcus aureus*). In vivo efficacy has been demonstrated in mice via intraperitoneal injection (2–4 mg/kg) [4,5] and oral administration (1.0–1.2 mg/kg feed) [6]. Sublancin also inhibits *Clostridium perfringens* (a Gram-positive bacteria) at a MIC of 8 μM, and controls necrotic enteritis in broilers in vivo at dose of 5.76 mg/L via water, which is much lower than that of lincomycin at 75 mg activity/L [7]. Beyond direct antimicrobial effects, it modulates the gut microbiota [8], enhances intestinal barrier function [5], and suppresses excessive inflammation [4]. Notably, it also potentiates innate immunity by upregulating host defense peptides [4,6,8,9]. These multifaceted properties, combined with undetectable drug residues (according to our pharmacokinetic studies in rats) and low resistance risk (consistent with other AMPs like nisin [10]), position sublancin as an ideal antibiotic alternative in livestock production, aligning with global antimicrobial stewardship goals [3,11,12,13].

Chronic toxicity tests evaluate long-term drug/toxin effects on animal health. They help determine the maximum no-observed-adverse-effect level (NOAEL) of a drug and are crucial for establishing a comprehensive safety profile, especially in assessing long-term impacts [14]. Chronic toxicity tests typically last for extended periods to simulate long-term drug exposure [14,15,16]. Tests are categorized by duration: subchronic (90-day) and chronic (180-day) toxicity tests. Tests are usually performed on specific animal models, such as C57 mice [14], Wistar rats [15], and Sprague–Dawley rats [16,17,18]. Various countries and regions have specific regulations and guidelines for chronic toxicity and carcinogenicity tests of veterinary drugs. Notable examples include guidelines from the US Environmental Protection Agency (USEPA) [19], the European Food Safety Authority (EFSA), and the United States Food and Drug Administration (FDA) [18]. To evaluate the safety of sublancin as a novel veterinary drug in practical applications, this study conducted 90-day subchronic and 180-day chronic toxicity tests in accordance with the Guidelines for Chronic Toxicity and Carcinogenicity Testing of Veterinary Drugs issued by the Ministry of Agriculture and Rural Affairs of the People’s Republic of China. In this study, varying doses of sublancin, equivalent to 1666–5000 times the dose shown to be efficacious in antibacterial function and immune enhancement, were administered to SD rats of different ages and sexes to systematically assess its effects on growth performance, hematological parameters, serum biochemistry, organ coefficients, and histopathology.

## 2. Materials and Methods

### 2.1. Chemicals and Reagents

Sublancin (purity > 93%), supplied by Sinagri Yingtai bio-peptide Co., Ltd. (Linzhou, Henan, China), has the following amino acid sequence: ESKICTWLGTGQCNCYK CKWEQSLQCRN.

### 2.2. Animals and Treatments

This study was approved by the Animal Care and Use Committee of China Agricultural University, which approved all the animal experiments in this study (approval number AW42800202-3-5). All the experimental protocols followed the guidelines for animal experiments provided by the Animal Care and Use Committee of China Agricultural University.

A total of 240 four-week-old male and female SPF-grade SD rats (70–90 g) were purchased from Beijing Weitonglihua Experimental Animal Technology Co., Ltd. (Beijing, China). For the subchronic (90-day) and chronic (180-day) toxicity tests, 80 and 160 rats were allocated, respectively. Rats were housed under standard conditions with a controlled temperature of 22–24 °C, humidity of 50–60%, and a light/dark cycle. All the rats had free access to a standard diet and drinking water throughout the experiment. On the basis of the acute toxicity test results (LD50 > 5000 mg/kg body weight) and regulatory guidelines (maximum 5% of feed weight) [20], we selected four dose levels: 0 mg/kg (control group), 2000 mg/kg (low-dose group), 10,000 mg/kg (medium-dose group), and 50,000 mg/kg (high-dose group). These doses represent 1666–5000 times the efficacious antibacterial dose (1.0–1.2 mg/kg) previously established in mice [6]. Each group consisted of 20 SD rats (10 males and 10 females), with the weight variation within each sex not exceeding 20% of the average body weight. For the subchronic toxicity test and chronic toxicity tests, the animals were organized into the same groups, with the number of animals doubled. Sublancin was added to the maintenance feed for rats at four dose levels: 0 mg/kg (control), 2000 mg/kg, 10,000 mg/kg, and 50,000 mg/kg. The feed was thoroughly mixed in a feed mixer to ensure uniform distribution and then pelleted. The maintenance feed was purchased from Beijing Keao Xieli Feed Co., Ltd. (Beijing, China).

### 2.3. Experiment 1: Observation and Measurement of Indicators of the Subchronic Toxicity Test for 90 Days

#### 2.3.1. General Observations

The overall health status of the experimental animals was monitored daily. Detailed records were kept of the rats’ behavior, including eye, mouth, and nose secretions; fecal consistency; signs of diarrhea or constipation; fecal color; and fur condition (e.g., dullness, piloerection). Each group of rats receiving a specific dose were closely monitored for toxicity signs, including the timing and appearance of symptoms. If an animal died or showed severe illness, immediate post-mortem examinations were performed. Detailed records were kept of any abnormal conditions and the time of death. Tissues from the heart, liver, spleen, lungs, kidneys, and duodenum were collected for histopathological analysis. Body weight and food intake were measured every five days, while water consumption was recorded every two days.

#### 2.3.2. Hematology

Blood samples were collected for hematological analysis from 5 males and 5 females in each experimental group on Day 45 (midpoint) and Day 90 (endpoint) of the study. The following hematological parameters were analyzed: hemoglobin (HGB), red blood cell count (RBC), white blood cell count (WBC), platelet count (PLT), hematocrit (HCT), eosinophils (EOS), basophils (BAS), monocytes (MO), and lymphocytes (LY).

#### 2.3.3. Serum Biochemical Parameters

Simultaneously with the hematological analysis, we assessed serum biochemical parameters, including albumin (ALB), alanine aminotransferase (ALT), aspartate aminotransferase (AST), blood urea nitrogen (BUN), total cholesterol (TC), creatinine (Cr), glucose (Glu), and total protein (TP).

#### 2.3.4. Pathological Examination

On Day 45 (midpoint) and Day 90 (endpoint) of the study, five male and five female rats from each experimental group were euthanized. A gross autopsy was then conducted. Any suspicious lesions observed were quickly fixed and preserved for histopathological analysis. During the gross examination, body and major organ weights were recorded, with organ-to-body weight ratios calculated for the heart, liver, kidneys, spleen, lungs, gastrointestinal tract, testes, and ovaries.

Following the gross autopsy, the heart, liver, spleen, lungs, kidneys, and duodenum were collected and fixed in a 10% neutral formalin solution. Routine paraffin-embedded sections were prepared and stained with hematoxylin and eosin (H&E) for a histopathological examination of these organs in both the high-dose and control groups. If lesions were observed, the corresponding organs from the lower-dose groups were also examined via histopathological analysis.

### 2.4. Experiment 2: Observation and Measurement of Indicators of the Chronic Toxicity Test for 180 Days

The experimental groups were organized similarly to Experiment 1, but with double the number of animals in each group. General observations were consistent with Experiment 1. However, histopathological assessments were expanded to the brain, testes, epididymis, ovaries, and uterus. Water intake was recorded every 5 days, consistent with the frequency of body weight and food intake measurements. Blood samples were collected on Days 60, 120, and 180 for hematological and biochemical analyses, following the same parameters as Experiment 1. On Days 60, 120, and 180 of the chronic toxicity studies, rats were sacrificed for necropsy, including a uterine assessment. Other indices and procedural steps were consistent with those in Experiment 1. Additionally, histopathological examinations of rat tissues included brain tissue, with other indices and procedural steps consistent with those in Experiment 1.

### 2.5. Statistical Analysis

Differences in food intake, water intake, daily weight gain, hematological and serum biochemical parameters, and organ coefficients between the treatment groups and negative controls were analyzed by *t*-tests. The data are presented as the mean ± SD. Incidence rates of organ lesions, tumors, and mortality were analyzed with chi-square (χ^2^) tests. We defined a *p*-value of less than 0.05 as statistically significant.

## 3. Results

### 3.1. Clinical Symptoms

In the subchronic (90-day) and chronic (180-day) toxicity studies, rats administered sublancin at three doses (2000 mg/kg, 10,000 mg/kg, and 50,000 mg/kg) showed normal behavior compared with the negative controls. All animals maintained normal physical conditions, including smooth fur without ocular, oral, or nasal discharge. Fecal characteristics (morphology and color) remained consistent across all groups, with no deviations from the typical light gray appearance observed in healthy rats.

### 3.2. Food Intake

**Figure 1:**
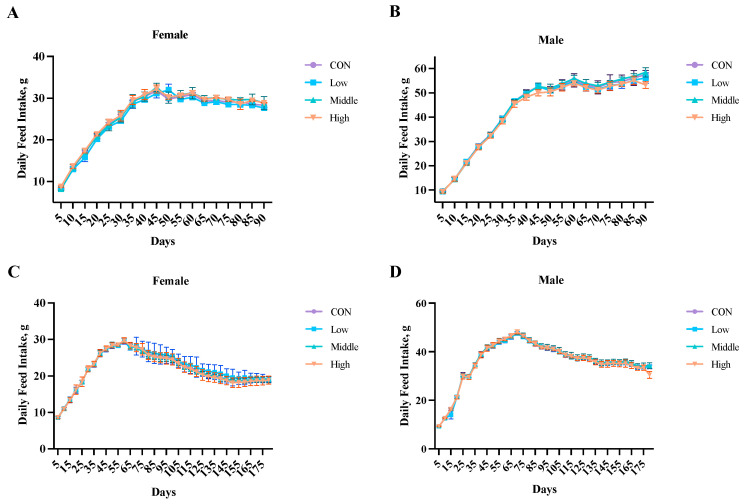
Effects of sublancin on the daily food intake of SD rats fed for the subchronic ((**A**,**B**); 90-day; *n* = 10/sex/group) and chronic ((**C**,**D**); 180-day; *n* = 20/sex/group) toxicity trials (g/day/rat). Each point represents the mean ± SD. CON group, basal diet; Low group, basal diet + 2000 mg/kg; Middle group, basal diet + 10,000 mg/kg; High group, basal diet + 50,000 mg/kg.

Statistical analysis of food intake (Figure 1; Tables S1 and S2-1–S2-3) revealed no significant differences (*p* > 0.05) between the control (CON) and sublancin-treated groups (2000, 10,000, or 50,000 mg/kg feed) in either the 90-day subchronic or 180-day chronic toxicity studies. This consistency was observed in both male and female rats, indicating that prolonged oral administration of sublancin at these doses did not alter feeding behavior.

### 3.3. Water Intake

**Figure 2:**
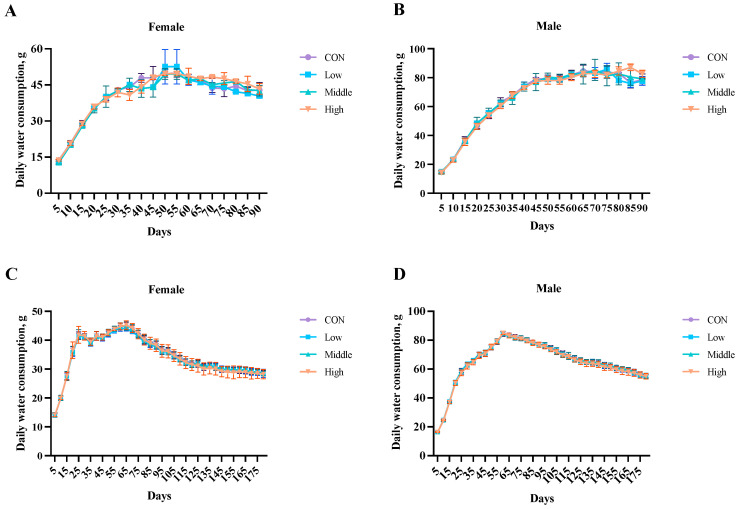
Effects of sublancin on the daily water intake of SD rats fed for the subchronic toxicity trial ((**A**,**B**); 90-day; *n* = 10/sex/group) and chronic ((**C**,**D**); 180-day; *n* = 20/sex/group) toxicity trials (g/day/rat). Each point represents the mean ± SD. CON group, basal diet; Low group, basal diet + 2000 mg/kg; Middle group, basal diet + 10,000 mg/kg; High group, basal diet + 50,000 mg/kg.

The water consumption analysis presented no significant differences (*p* > 0.05) between the control and sublancin-treated groups (2000–50,000 mg/kg feed) in either the 90-day subchronic or 180-day chronic toxicity studies (Figure 2; Tables S3 and S4-1–S4-3). These findings demonstrate that prolonged ad libitum administration of sublancin at these doses (2000 mg/kg, 10,000 mg/kg, and 50,000 mg/kg of feed) did not affect water intake in rats of either sex.

### 3.4. Average Daily Weight Gain

**Figure 3:**
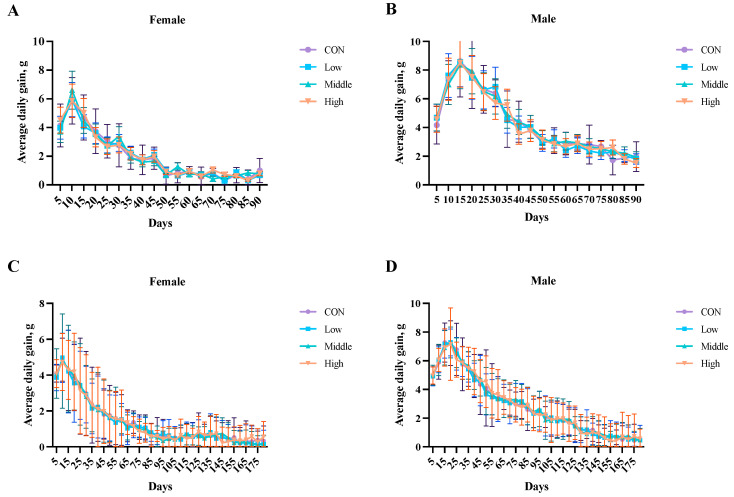
Effects of sublancin on the average daily gain of SD rats fed for the subchronic ((**A**,**B**); 90-day; *n* = 10/sex/group) and chronic ((**C**,**D**); 180-day; *n* = 20/sex/group) toxicity trials (g/day/rat). Each point represents the mean ± SD. CON group, basal diet; Low group, basal diet + 2000 mg/kg; Middle group, basal diet + 10,000 mg/kg; High group, basal diet + 50,000 mg/kg.

The body weight gain analysis (Figure 3; Tables S5 and S6-1–S6-3) showed no significant differences (*p* > 0.05) between the control and sublancin-treated groups (2000–50,000 mg/kg feed) during the 90-day subchronic or 180-day chronic toxicity studies. These findings indicate that continuous exposure to sublancin at these doses ranging from 2000 to 50,000 mg/kg of feed did not affect growth performance in either male or female rats.

### 3.5. Hematological Parameters

**Figure 4:**
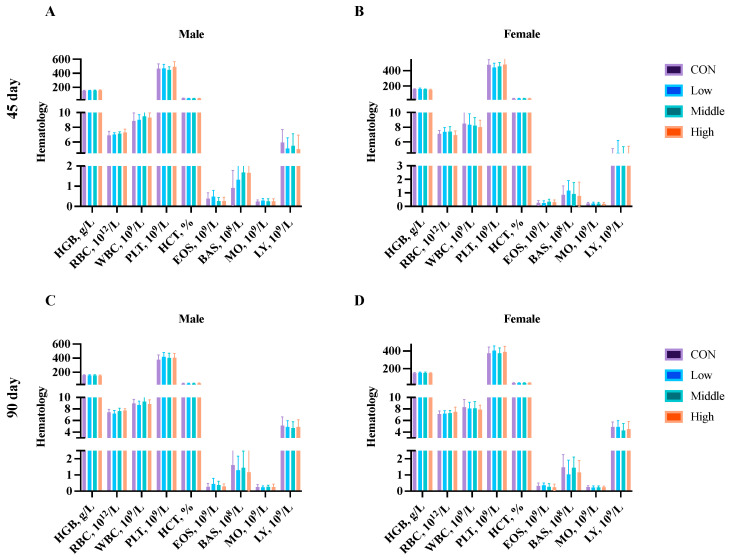
Effects of sublancin on the hematology of SD rats fed for 45 (**A**,**B**) days and 90 (**C**,**D**) days during the subchronic toxicity trial (*n* = 5/sex/group). Each point represents the mean ± SD. CON group, basal diet; Low group, basal diet + 2000 mg/kg; Middle group, basal diet + 10,000 mg/kg; High group, basal diet + 50,000 mg/kg. HGB, hemoglobin; RBC, red blood cell count; WBC, white blood cell count; PLT, platelets; HCT, hematocrit; EOS, eosinophils; BAS, basophils; MO, monocytes; LY, lymphocytes.

**Figure 5:**
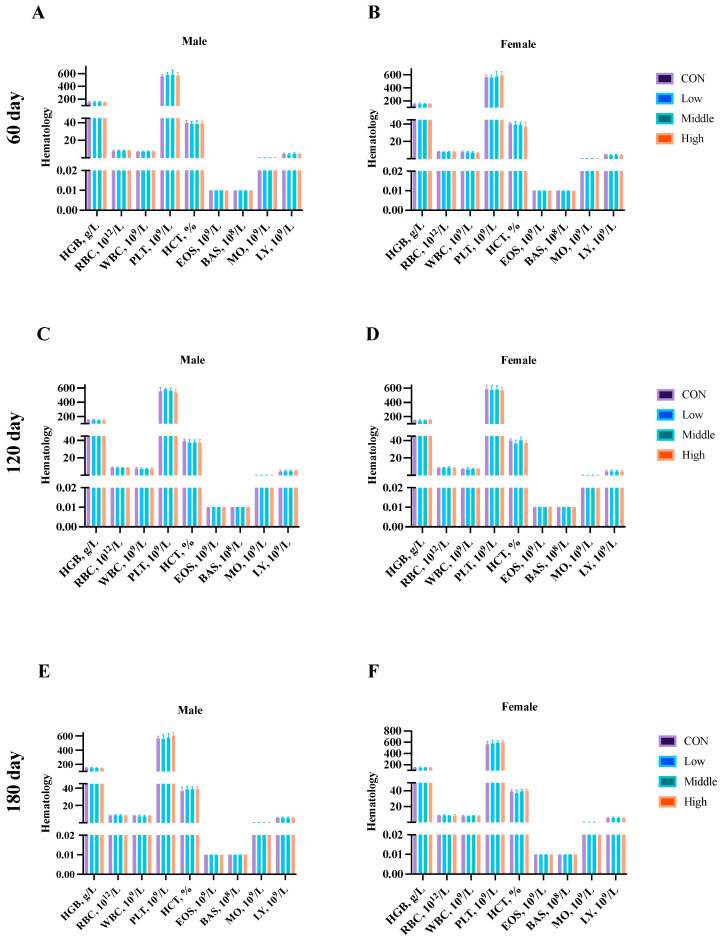
Effects of sublancin on the hematology of SD rats fed for 60 (**A**,**B**), 120 (**C**,**D**) and 180 (**E**,**F**) days during the chronic toxicity trial (*n* = 5/sex/group). Each point represents the mean ± SD. CON group, basal diet; Low group, basal diet + 2000 mg/kg; Middle group, basal diet + 10,000 mg/kg; High group, basal diet + 50,000 mg/kg. HGB, hemoglobin; RBC, red blood cell count; WBC, white blood cell count; PLT, platelet count; HCT, hematocrit; EOS, eosinophils; BAS, basophils; MO, monocytes; LY, lymphocytes.

Hematological analysis was performed on blood samples collected from five male and five female rats per group at (1) Days 45 and 90 (subchronic study endpoint), and (2) Days 60, 120, and 180 (chronic study endpoint). Analysis of the key parameters (HGB, RBC, WBC, PLT, HCT, EOS, BAS, MO, and LY) revealed no significant differences (*p* > 0.05) between the control and sublancin-treated groups (2000–50,000 mg/kg feed) at any time point (Figure 4 and Figure 5; Tables S7 and S8). These findings demonstrate that prolonged sublancin exposure at these doses did not induce hematological alterations in either sex.

### 3.6. Clinical Biochemistry

**Figure 6:**
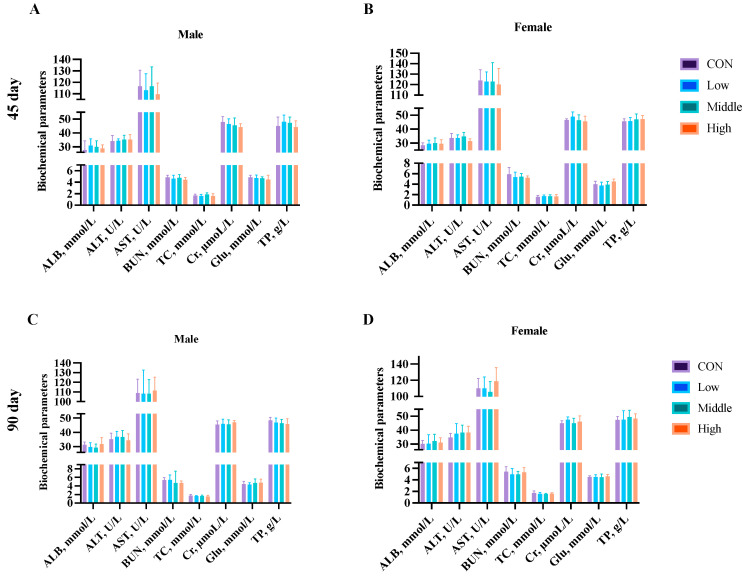
Effects of sublancin on the biochemical parameters of SD rats fed for 45 (**A**,**B**) and 90 (**C**,**D**) days during the subchronic toxicity trial (*n* = 5/sex/group). Each point represents the mean ± SD. The data were compared for statistical significance with the same-sex control group. CON group, basal diet; low group, basal diet + 2000 mg/kg; middle group, basal diet + 10,000 mg/kg; high group, basal diet + 50,000 mg/kg. ALB, albumin; ALT, alanine aminotransferase; AST, aspartate aminotransferase; BUN, blood urea nitrogen; TC, total cholesterol; Cr, creatinine; Glu, glucose; TP, total protein.

**Figure 7:**
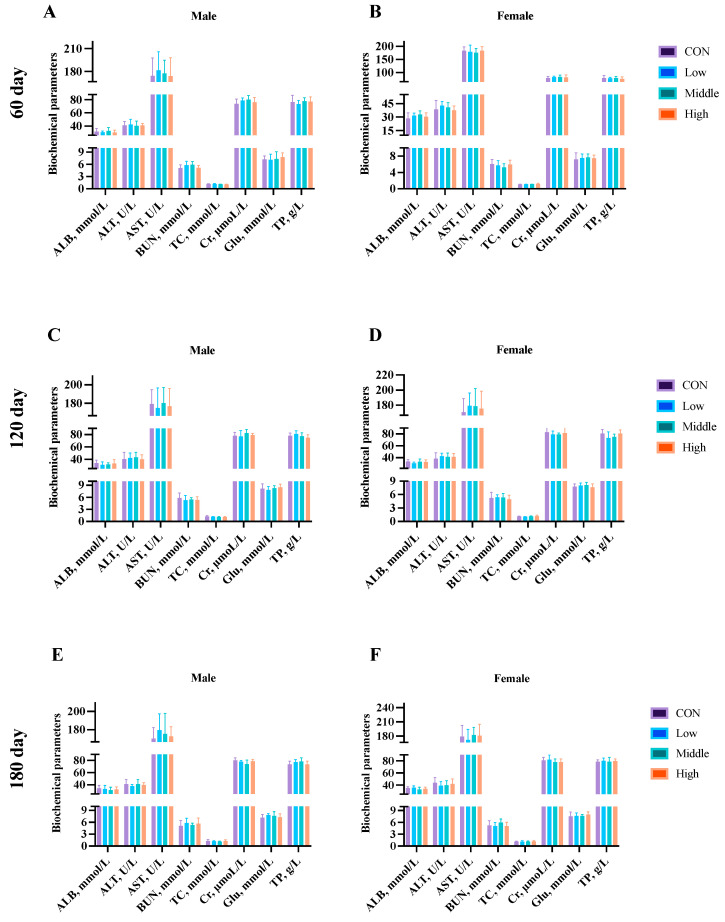
Effects of sublancin on the serum biochemical parameters of SD rats fed for 60 (**A**,**B**), 120 (**C**,**D**) and 180 (**E**,**F**) days during the chronic toxicity trial (*n* = 5/sex/group). Each point represents the mean ± SD. CON group, basal diet; Low group, basal diet + 2000 mg/kg; Middle group, basal diet + 10,000 mg/kg; High group, basal diet + 50,000 mg/kg. ALB, albumin; ALT, alanine aminotransferase; AST, aspartate aminotransferase; BUN, blood urea nitrogen; TC, total cholesterol; Cr, creatinine; Glu, glucose; TP, total protein.

Blood biochemical analysis was conducted at multiple time points: Day 45 (mid-study) and Day 90 (endpoint) in the subchronic study, and Days 60, 120, and 180 (endpoint) in the chronic study. Evaluation of the key parameters (ALB, ALT, AST, BUN, TC, Cr, Glu, and TP) revealed no significant differences (*p* > 0.05) between the control and sublancin-treated groups (2000–50,000 mg/kg feed) at any time point (Figure 6 and Figure 7; Tables S9 and S10). These results demonstrate that prolonged dietary exposure to sublancin at these doses did not induce clinically relevant alterations in blood biochemistry in either male or female rats.

### 3.7. Organ Index

**Figure 8:**
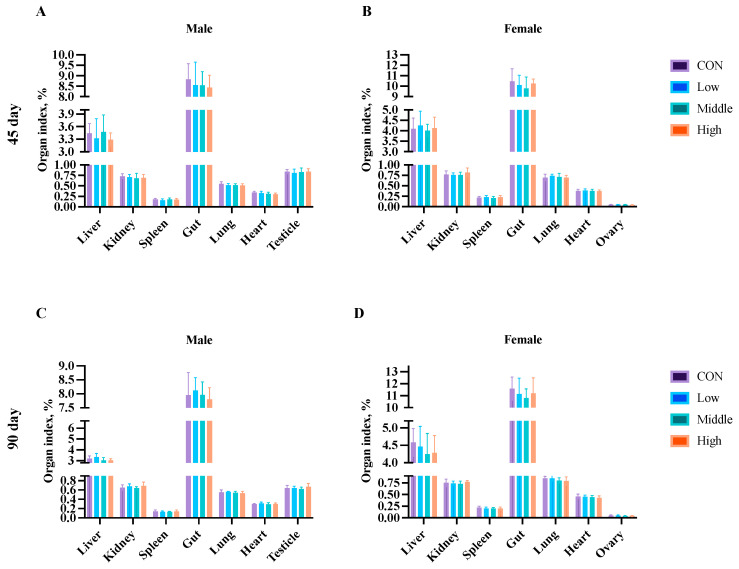
Effects of sublancin on the organ index of SD rats fed for 45 (**A**,**B**) and 90 (**C**,**D**) days during the subchronic toxicity trial (*n* = 5/sex/group). Each point represents the mean ± SD. CON group, basal diet; Low group, basal diet + 2000 mg/kg; Middle group, basal diet + 10,000 mg/kg; High group, basal diet + 50,000 mg/kg.

**Figure 9:**
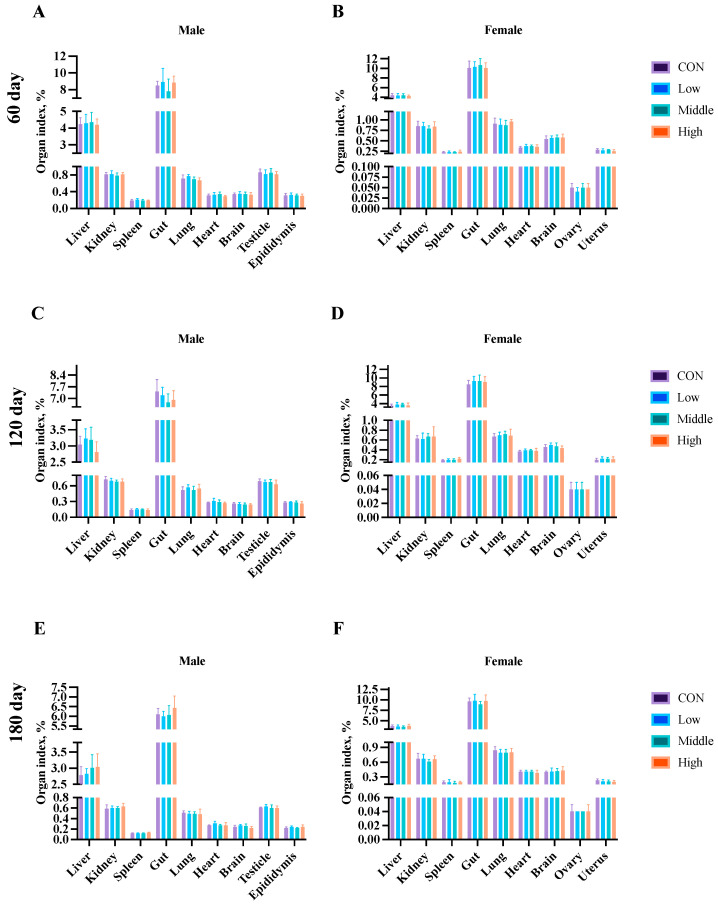
Effects of sublancin on the organ index of SD rats fed for 60 (**A**,**B**), 120 (**C**,**D**) and 180 (**E**,**F**) days during the chronic toxicity trial (*n* = 5/sex/group). Each point represents the mean ± SD. CON group, basal diet; Low group, basal diet + 2000 mg/kg; Middle group, basal diet + 10,000 mg/kg; High group, basal diet + 50,000 mg/kg.

Organ weight analysis was performed at the subchronic study’s midpoint (Day 45) and endpoint (Day 90), and during the chronic study (Days 60, 120, and 180) using five rats per sex per group. No significant differences (*p* > 0.05) were observed in relative organ weights (heart, liver, kidneys, spleen, lungs, gastrointestinal tract, brain, and reproductive organs) between the control and sublancin-treated groups (2000–50,000 mg/kg feed) at any time point (Figure 8 and Figure 9; Tables S11 and S12). These findings demonstrate that chronic ad libitum exposure for up to 180 days to sublancin at doses from 2000 to 50,000 mg/kg of feed did not induce organotoxicity (including the heart, liver, kidneys, spleen, lungs, gastrointestinal tract, brain, testes, epididymides, ovaries, and uterus) at the tested doses.

### 3.8. Histopathology Analysis

Throughout the 90-day subchronic and 180-day chronic toxicity studies, no treatment-related clinical signs or mortality were observed in any experimental groups. The gross necropsy examination revealed no abnormalities in either the sublancin-treated (2000–50,000 mg/kg feed) or control groups.

Histopathological analysis was conducted on the key organs (heart, liver, spleen, lungs, kidneys, duodenum, brain, and reproductive organs) from the high-dose (50,000 mg/kg) and control groups at the study’s termination. A comprehensive evaluation of multiple parameters, including clinical observations, body weight, food/water consumption, hematology, clinical biochemistry, organ weights, and gross pathology, revealed no significant treatment-related effects (*p* > 0.05). Consequently, the histopathological comparison focused on the highest-dose group versus the controls.

**Figure 10:**
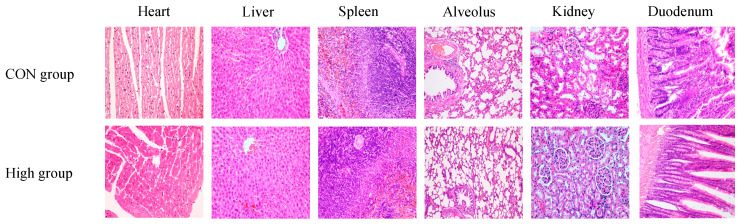
Histological sections of various tissues (heart, liver, spleen, alveolus, kidneys, and duodenum) from SD rats subjected to 90-day subchronic toxicity testing with sublancin (H&E staining, 40×). CON group, basal diet; High group, basal diet + 50,000 mg/kg.

**Figure 11:**
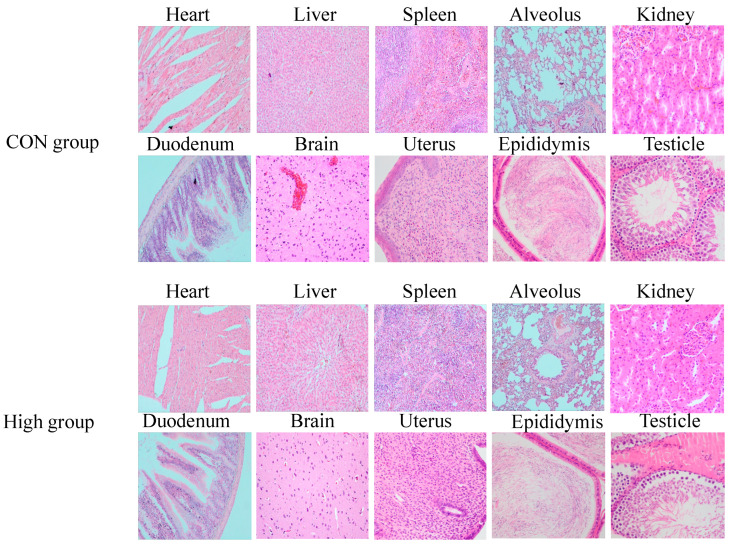
Histological sections of various tissues (heart, liver, spleen, alveolus, kidneys, duodenum, brain, and reproductive tissues) from SD rats subjected to 180-day chronic toxicity testing with sublancin (H&E staining, 40×). CON group, basal diet; High group, basal diet + 50,000 mg/kg.

As shown in Figure 10 and Figure 11, microscopic examination demonstrated a normal tissue architecture in all examined organs, with no pathological alterations attributable to sublancin administration. These results conclusively demonstrate that chronic ad libitum exposure to sublancin at doses up to 50,000 mg/kg feed induced no adverse effects on major organ systems in SD rats.

In summary, administering sublancin at doses ranging from 2000 to 50,000 mg/kg of feed did not cause significant differences in appetite, water intake, or weight gain in SD rats (*p* > 0.05). Additionally, no significant differences were observed in hematological, biochemical, or pathological parameters in the sublancin-treated groups when compared with the control group (*p* > 0.05). These results indicate that sublancin does not exhibit subchronic and chronic toxicity within the dose range of 2000 to 50,000 mg/kg of feed. The NOEL was established at 50,000 mg/kg of feed based on both the 90-day subchronic and 180-day chronic toxicity studies.

## 4. Discussion

As antimicrobial resistance increasingly threatens human health, developing alternative compounds to replace conventional antibiotics has become a research imperative [21,22]. Antimicrobial peptides (AMPs) have broad-spectrum antimicrobial activity. They are highly effective against bacteria, fungi, parasites, and viruses, while showing a low tendency for developing resistance [23,24]. The unique pharmacokinetics and bactericidal mechanisms of AMPs position them as potential candidates to replace conventional antibiotics [25]. In animal husbandry, AMPs are recognized as ideal antibiotic alternatives [26], while in human medicine, they represent promising next-generation therapeutic agents for infectious diseases [27]. Sublancin is a cyclic (glycol) peptide made up of 37 amino acids with a molecular weight of 3877.642 Da [11]. The unique α-helical secondary structure has been confirmed through circular dichroism (CD) and nuclear magnetic resonance (NMR) studies [28,29]. Studies demonstrate that sublancin not only effectively inhibits the growth of diverse Gram-positive bacteria (e.g., *Staphylococcus aureus* and *Clostridium perfringens*) in vitro and in vivo [4,5,6,7], but also confers broad-spectrum protection against multidrug-resistant pathogen infections [8,30,31]. Given its unique antibacterial mechanism as an AMP and low resistance potential, sublancin may represent a promising candidate for innovative veterinary therapeutics [12,13].

In China, veterinary drug safety evaluations strictly adhere to the Regulations on Veterinary Drug Administration and Measures for Veterinary Drug Registration, in accordance with the Guidelines for Safety Pharmacology Studies of Veterinary Chemical Drugs issued by the Ministry of Agriculture and Rural Affairs (MARA). These regulations mandate that novel veterinary compounds (e.g., antibiotic alternatives) must undergo systematic acute, subchronic, and chronic toxicity testing, with particular emphasis on assessing the long-term effects on animal growth, hematology, organ function, and histopathology (MARA Announcement No. 1247). Our toxicology study evaluated sublancin using standardized 90-day and 180-day testing protocols at doses up to 50,000 mg/kg feed. This maximum dose represented 4166–5000× the established efficacious dose in rats (1.0–1.2 mg/kg) [6] and 83× the effective dose in broilers (equivalent to 60 mg/kg feed) [9] in vivo. The study design complied with GLP standards and regulatory requirements for veterinary drug registration.

Before initiating clinical trials to evaluate the efficacy of AMPs, acute and chronic toxicity tests in animal models are essential prerequisites. Chronic toxicity testing is crucial for establishing a comprehensive safety profile of investigational drugs, with particular emphasis on characterizing the long-term effects [14]. Subchronic toxicity studies, typically conducted over 90 days, provide detailed data on compound-induced changes in growth, development, physiological functions, and organ pathology [32]. These studies identify potential chronic toxicities and ensure that veterinary drugs do not significantly compromise animal health in practical applications [32]. Specifically, they determine the toxicological threshold and safe dosage by monitoring behavioral anomalies, appetite, water consumption, weight gain dynamics, hematological profiles, serum biochemistry, and organ coefficients, reflecting the potential impacts on fundamental physiological functions [33]. A 90-day trial identifies the maximum dose that does not significantly harm animals, providing critical information for subsequent chronic toxicity studies [34]. Subchronic toxicity studies constitute a pivotal element in drug safety evaluation, forming the foundation for nonclinical safety assessments and serving as a mandatory prerequisite prior to a clinical trial’s initiation [32]. Chronic toxicity studies systematically characterize 180-day exposure effects in animals, focusing on chronic disease progression, parenchymal organ damage (liver, kidneys, and heart), and potential carcinogenicity. They determine the NOEL for long-term drug use, establishing a scientific basis for safe dosages [17,32]. These systematic toxicity tests ensures the safety of veterinary drugs used in practical applications and comply with national and international regulations [35]. Given China’s policies prohibiting antibiotics in animal feed and limiting their use in veterinary medicine, this study’s results are significant for developing and promoting novel veterinary drugs without residues [1]. These systematic toxicological evaluations provide essential data for establishing safety standards of investigational veterinary drugs, while supporting regulatory compliance with national and international guidelines for veterinary pharmaceutical development.

Assessing the safety of potential veterinary drugs is crucial for regulatory approval and usually involves a thorough review of various scientific documents, including monographs, peer-reviewed articles, and reports. This study investigates the safety of sublancin as a potential antibiotic alternative in rats, especially examining its long-term chronic toxicity to address a gap in the existing literature. Following the Guidelines for Chronic Toxicity and Carcinogenicity Testing of Veterinary Drugs from the Ministry of Agriculture and Rural Affairs of the People’s Republic of China, we performed 90-day subchronic and 180-day chronic toxicity tests on SD rats with varying doses of sublancin to comprehensively assess its long-term feeding effects. The results indicated that sublancin, administered at doses of 2000–50,000 mg/kg feed, had no significant adverse effects on the growth and development, hematological parameters, serum biochemical parameters, or organ coefficients of rats, demonstrating a favorable safety profile. The trial involved continuous administration of the test substance for 60, 120, and 180 days. During this period, researchers observed adverse reactions and other indicators in the animals to assess the toxicity level and identify potential target organs. The evaluation criteria included changes in the food intake, water consumption, and body weight of the test animals, along with alterations in physiological and biochemical parameters and organ indices, and histopathological changes [17]. Neither the subchronic nor chronic toxicity tests revealed any abnormalities in the mental status, excreta, or diet of rats across all dose groups, and there were no recorded cases of poisoning or death. Collectively, these findings demonstrate sublancin’s long-term safety profile and support its translational development as a veterinary therapeutic.

During both the 90-day and 180-day trials, sublancin did not negatively affect key physiological parameters like appetite, water consumption, or weight gain in rats, indicating that it does not alter the basic physiological functions of animals. Additionally, long-term feeding of sublancin to rats did not result in significant differences in appearance, behavior, or mental state compared with the control group, confirming that sublancin has no adverse effects on the daily behavior or quality of life of rats. Changes in body weight are among the most significant indicators of toxicity in animals [35]. Existing literature suggests that an initial weight loss of 10% or more in experimental animals may be a strong indicator of adverse side effects and could potentially endanger survival. In contrast, our study revealed no statistically significant variations in the body weight gain of rats. Although there are no direct reports on sublancin’s effects on animal growth and production performance, numerous studies have demonstrated that adding various doses of AMPs to feed significantly promotes growth and improves production performance in broiler chickens [36,37], laying hens [38], weaned piglets [39], aquaculture [40,41], and ruminants [42]. Integrating all endpoints, administration of sublancin at doses up to 50,000 mg/kg demonstrated no significant alterations in fundamental physiological parameters, including appetite, water intake, and body weight dynamics, thereby establishing a potentially wide safety margin based upon efficacy data from the literature (the NOEL here was 50,000 mg/kg) for maintaining homeostatic growth and metabolic functions during chronic 180-day exposure.

Food intake and water consumption are important indicators of animal health. Systematic recording regularly enables prompt detection of behavioral abnormalities and variations in physiological parameters. If persistent abnormal changes in food intake or water consumption occur, particularly those independent of environmental factors or physiological conditions, the possibility of chronic toxicity must be systematically evaluated. Sublancin showed no significant effects on the water and food intake of rats. This finding provides evidence to support the long-term safety of sublancin in rodent models and support further translational development. In summary, AMPs promote livestock growth through direct growth-promoting effects and indirect effects by enhancing overall animal health and welfare, supporting their potential application in livestock growth.

Hematology and blood indices reflect the animal’s ability to digest and absorb nutrients, its nutrient metabolism, and overall health. Monitoring these parameter changes enables a more precise toxicity evaluation of the test substance [43]. In this study, sublancin-treated rats showed no significant differences in hematological parameters (hemoglobin, red blood cell count, white blood cell count, and platelet count) compared with the control group, demonstrating no adverse effects on hematopoietic function. Furthermore, the liver function indicators (ALT, AST, ALP), kidney function (BUN, Cr), and blood lipid levels (TCH, TG) remained unaffected by sublancin, suggesting a low toxicity risk to the rats’ major metabolic organs and systems. Published studies demonstrate that sublancin exerts significant therapeutic effects in animal models of specific pathological conditions. Wang et al. [12] demonstrated that oral sublancin administration (1 mg/kg) significantly upregulated *IL-1*, *IL-6*, and *TNF-α* gene expression in the macrophages of cyclophosphamide-induced immunosuppressed mice. In a murine methicillin-resistant *Staphylococcus aureus* (MRSA) model, sublancin exhibited anti-inflammatory activity, elevating chemokines (CXCL1 and MCP-1) and suppressing the production of pro-inflammatory cytokines (TNF-α) [8]. Wang et al. [7] further demonstrated that dietary sublancin supplementation reduced pro-inflammatory cytokines (IL-1β, IL-6, and TNF-α) in the ileum of broiler chickens with *Clostridium perfringens*-induced necrotic enteritis, confirming its anti-inflammatory efficacy. Liu et al. [9] also found that dietary sublancin significantly enhanced the immune response in specific-pathogen-free broiler chickens vaccinated against Newcastle disease, promoting lymphocyte proliferation; stimulating secretion of IFN-γ, IL-10, and IL-4; and elevating antibody titers. Notably, these effects were more pronounced at lower doses, suggesting its potential as a vaccine adjuvant. Overall, the high doses of sublancin in this study did not cause changes in serum biochemical parameters, indicating a low toxicity risk to the major metabolic organs and systems of animals under normal physiological conditions. Furthermore, the existing literature reports that sublancin has significant therapeutic effects on test animals under pathological conditions [9,12], further confirming its safety and providing scientific evidence for its therapeutic potential in practical applications.

Measuring organ coefficients serves as a sensitive indicator of a drug’s impact on organs, and their fluctuations are recognized as markers for evaluating the effects of drug exposure on organ integrity [44]. This study found no significant differences in the organ coefficients of the heart, liver, spleen, lungs, and kidneys between sublancin-treated rats and the control group, indicating that sublancin does not cause significant damage or hypertrophy in these organs. Furthermore, the histopathological examination revealed no abnormal structural changes in the major organs of sublancin-treated rats, further confirming the safety of sublancin for these organs. Additionally, previous studies indicate that sublancin has protective and reparative effects on animal organs under pathological conditions. Wang et al. [5] found that adding sublancin to feed reduces intestinal damage caused by *Staphylococcus aureus* infection in young mice. Specifically, administering 4 mg/kg sublancin significantly improved intestinal repair and reduced mortality. Wang et al. [7] reported that dietary sublancin supplementation significantly increased jejunal villus height and duodenal crypt depth in broiler chickens with *Clostridium perfringens*-induced necrotic enteritis. Histopathological and functional analyses revealed no sublancin-induced abnormalities in renal structure or function, even at the maximum tested dose (50,000 mg/kg), despite kidneys being the principal detoxification organs [45]. Further histopathological examination revealed no signs of toxicity in other organs. Morphological observations of the major organs in the rats showed no necrosis, inflammation, or other pathological changes. This finding further demonstrates that high doses of sublancin have no subchronic toxicity and are considered highly safe. Collectively, the integrated subchronic (90-day) and chronic (180-day) toxicity data establish a NOEL of 50,000 mg/kg for sublancin, whichm together with the previously determined acute oral LD50 exceeding 5000 mg/kg, demonstrates systemic and organ-level safety across all evaluated endpoints, when considered against published efficacious dosing levels both in mice [6] and broilers [7,9]. These robust toxicological findings provide compelling experimental evidence to support further the preclinical development of sublancin as a veterinary therapeutic candidate.

It is noteworthy that published studies on sublancin administration in animal models have employed either intraperitoneal injection [4,5] or dietary/water supplementation [9,12]. As a peptide antimicrobial, sublancin shares the common delivery challenges of oral peptides [46], though its actual gastrointestinal stability needs empirical determination. While intraperitoneal injection introduces compounds directly into systemic circulation, risking acute toxicity from localized high concentrations (e.g., peritoneal irritation) [47], oral administration better mimics real-world exposure through gastrointestinal absorption [48]. Actually, our GLP-compliant study demonstrated an exceptional safety profile for sublancin: even at 50,000 mg/kg orally, no treatment-related toxicity was observed. Specifically, we found the absence of gastrointestinal symptoms, no clinically significant hematological changes, and normal histopathology scores. These findings, consistent with our following study of sublancin’s gastric stability in SD rats, suggest that sublancin maintains structural integrity during digestion, with its degradation products exhibiting minimal toxic potential. Our completed pharmacokinetic studies in rats (data to be published) further support that oral administration of sublancin to rats results in minimal systemic absorption, primarily localizes in the gastrointestinal tract, and undergoes rapid excretion without evidence of tissue accumulation or plasma protein binding. The PK profile suggests that sublancin may retain partial stability in the gastrointestinal tract. Future investigations comparing PK/PD parameters across different administration routes may further optimize the dosing strategies.

## 5. Conclusions

This study systematically evaluated the safety of sublancin as a novel veterinary drug candidate through 90-day subchronic and 180-day chronic oral toxicity tests at doses of 2000, 10,000, and 50,000 mg/kg in feed. Comprehensive assessments revealed no adverse effects on physiological parameters (appetite, weight gain, water consumption), hematological indices, serum biochemistry, or organ histopathology in SD rats, establishing a NOEL of 50,000 mg/kg. These findings clearly support the long-term safety of oral administration of sublancin and its potential development as a veterinary therapeutic.

## Data Availability

Data are available upon request.

## Supplementary Materials

The following supporting information can be downloaded at https://www.mdpi.com/article/10.3390/toxics13050413/s1. Table S1. Effects of sublancin on the daily food intake of rats fed for 45 and 90 days during the subchronic toxicity trial. Table S2-1. Effects of sublancin on the daily food intake of rats fed for 60 days during the chronic toxicity trial. Table S2-2. Effect of sublancin on the daily food intake of rats fed for 120 days during the chronic toxicity trial. Table S2-3. Effect of sublancin on the daily food intake of rats fed for 180 days during the chronic toxicity trial. Table S3. Effect of sublancin on the daily water intake of rats fed for 45 and 90 days during the subchronic toxicity trial. Table S4-1. Effect of sublancin on the daily water intake of rats fed for 60 days during the chronic toxicity trial. Table S4-2. Effect of sublancin on the daily water intake of rats fed for 120 days during the chronic toxicity trial. Table S4-3. Effect of sublancin on the daily water intake of rats fed for 180 days during the chronic toxicity trial. Table S5. Effect of sublancin on the average daily gain of rats fed for 45 and 90 days during the subchronic toxicity trial. Table S6-1. Effect of sublancin on the average daily gain of rats fed for the 60 days during chronic toxicity trial. Table S6-2. Effect of sublancin on the average daily gain of rats fed for 120 days during the chronic toxicity trial. Table S6-3. Effect of sublancin on the average daily gain of rats fed for 180 days during the chronic toxicity trial. Table S7. Effect of sublancin on the hematology of rats fed for 45 and 90 days during the subchronic toxicity trial. Table S8. Effect of sublancin on the hematology of rats fed for 60, 120, and 180 days during the chronic toxicity trial. Table S9. Effect of sublancin on the biochemical parameters of rats fed for 45 and 90 days during the subchronic toxicity trial. Table S10. Effect of sublancin on the serum biochemical parameters of rats fed for 60, 120, and 180 days during the chronic toxicity trial. Table S11. Effect of sublancin on the organ index of rats fed for 45 and 90 days during the subchronic toxicity trial. Table S12. Effect of sublancin on the organ index of rats fed for 60, 120, and 180 days during the chronic toxicity trial.

## Abbreviations

The following abbreviations are used in this manuscript.

SD	Sprague–Dawley
NOEL	No-observed-effect level
NOAEL	No-observed-adverse-effect level
LD	Linear dichroism
E	Glutamic acid
S	Serine
K	Lysine
I	Isoleucine
C	Cysteine
T	Threonine
W	Tryptophan
L	Leucine
G	Glycine
T	Threonine
Q	Glutamine
N	Asparagine
Y	Tyrosine
R	Arginine
N	Asparagine
HGB	Hemoglobin
RBC	Red blood cell count
WBC	White blood cell count
PLT	Platelet count
HCT	Hematocrit
EOS	Eosinophils
BAS	Basophils
MO	Monocytes
LY	Lymphocytes
TP	Total protein
ALB	Albumin
ALT	Alanine aminotransferase
AST	Aspartate aminotransferase
BUN	Blood urea nitrogen
TC	Total cholesterol
Cr	Creatinine
Glu	Glucose
TG	Triglycerides
H&E	Hematoxylin and eosin
AMPs	Antimicrobial peptides
