# Supplementary material for: Subchronic and Chronic Toxicity Assessment of Sublancin in Sprague–Dawley Rats
Source: Toxics. 2025 May 21;13(5):413. doi: 10.3390/toxics13050413 (PMC12115613; doi:10.3390/toxics13050413)
Supplement: Supplementary file 1 [file toxics-13-00413-s001.zip › toxics-3510677-supplementary.pdf]

Evaluation of Subchronic and Chronic Toxicity of Sublancin: A 90-Day and 180-Day Study in SD Rats

Table S1. Effects of Sublancin on the daily feed intake of SD rats fed for 45 and 90 days during subchronic toxicity trial.

Groups		CON		Low		Middle		High	
Experimental rats		♀ 10	♂ 10	♀ 10	♂ 10	♀ 10	♂ 10	♀ 10	♂ 10
Average food intake of rats during each experimental period (g/day/rat)	1-5 day	8.56±0.36	9.62±0.41	8.24±0.17	9.48±0.12	8.21±0.40	9.59±0.54	8.81±0.20	9.32±0.61
	6-10 day	13.32±0.27	14.45±0.63	12.99±0.36	14.57±0.80	13.40±0.66	14.43±0.90	13.68±0.49	14.65±0.99
	11-15 day	16.94±0.43	21.67±0.85	15.80±1.02	21.68±0.33	17.15±0.60	21.18±0.85	17.44±0.58	21.15±0.80
	16-20 day	20.94±0.58	28.19±0.83	20.20±0.52	27.85±0.32	20.83±0.54	27.95±0.90	21.38±0.41	27.61±1.11
	21-25 day	23.87±0.25	32.93±0.95	23.16±0.32	32.76±0.55	23.51±1.31	32.69±1.14	24.26±0.70	32.38±0.97
	26-30 day	25.62±0.58	39.45±1.05	24.56±0.59	39.40±0.78	25.50±1.20	38.99±0.85	25.81±1.28	38.40±1.19
	31-35 day	29.18±0.23	46.54±0.70	28.59±1.04	46.60±0.21	29.29±1.62	46.36±0.97	29.57±1.10	45.42±1.34
	36-40 day	30.46±0.27	50.08±1.20	29.66±0.58	49.57±0.48	30.41±1.13	49.64±1.41	30.87±1.20	48.27±1.26
	41-45 day	31.77±0.26	52.55±0.75	31.17±1.07	52.65±1.43	32.60±0.99	52.65±1.43	32.19±0.96	50.02±1.27
Average daily food intake of rats (g/day/rat)		22.30±0.36	32.83±0.82	21.17±1.00	32.73±0.56	22.20±0.95	29.67±1.39	22.60±0.77	32.02±1.06
Average food intake of rats during each experimental period (g/day/rat)	46-50 day	29.80±0.23	51.31±1.43	31.99±1.37	50.92±0.34	30.14±1.35	51.89±1.71	30.27±0.53	50.40±1.69
	51-55 day	30.33±0.51	53.33±2.11	29.73±0.22	52.99±0.29	30.84±0.73	53.81±1.36	30.68±0.44	52.32±1.54
	56-60 day	30.91±0.42	55.17±2.71	30.25±0.63	54.53±0.93	31.25±0.49	55.86±1.43	31.25±0.55	54.02±1.12
	61-65 day	29.27±0.28	53.19±2.39	28.82±0.13	52.52±0.54	29.93±0.86	53.89±1.65	29.69±0.31	52.16±1.33
	66-70 day	29.46±0.55	52.30±2.72	29.15±0.67	51.43±0.43	30.10±0.61	52.91±1.41	30.14±0.03	51.16±1.20
	71-75 day	29.04±0.54	54.24±3.25	28.53±0.01	53.06±1.48	29.67±0.41	54.40±0.73	29.76±0.48	52.83±1.78
	76-80 day	29.11±0.48	54.63±2.70	28.47±0.59	53.64±1.74	29.64±0.50	55.72±0.80	28.57±1.24	53.64±0.91
	81-85 day	28.61±0.09	56.15±3.07	28.28±0.22	55.20±1.48	29.70±1.30	56.77±1.09	29.48±0.16	55.15±1.70
	86-90 day	28.28±0.58	57.51±1.79	27.65±0.26	56.13±2.14	28.83±1.55	58.45±1.90	28.88±0.05	56.48±1.26
Average daily food intake of rat (g/day/rat)		29.42±0.41	54.20±2.46	29.21±0.79	53.38±1.04	30.01±0.80	54.85±1.34	29.86±0.42	53.30±1.39

Note: The data were compared for statistical significance with the same-sex control group. In the same row, values with different small letter superscripts mean significant difference (P<0.05), while with the same or no letter superscripts mean no significant difference (P>0.05). The same as below. CON group, basal diet; Low group, basal diet + 2000 mg/kg; Middle group, basal diet + 10000 mg/kg. High group, basal diet + 50000 mg/kg.

Table S2-1. Effects of sublancin on the daily food intake of SD rats fed for 60 days during chronic toxicity trial.

Groups		CON		Low		Middle		High	
Experimental rats		♀ 20	♂ 20	♀ 20	♂ 20	♀ 20	♂ 20	♀ 20	♂ 20
Average food intake of rats during each experimental period (g/day/rat)	1-5 day	8.57±0.32	9.31±0.21	8.60±0.28	9.30±0.22	8.58±0.34	9.35±0.16	8.66±0.38	9.33±0.17
	6-10 day	10.96±0.34	12.58±0.36	11.10±0.36	12.65±0.33	10.94±0.40	12.69±0.26	10.99±0.30	12.68±0.18
	11-15 day	13.39±0.70	16.44±0.55	13.54±0.71	14.00±1.63	13.50±0.64	16.39±0.35	13.44±0.63	16.39±0.42
	16-20 day	16.05±0.77	21.39±0.58	16.11±0.83	21.43±0.30	16.23±0.58	21.35±0.55	16.26±1.29	21.29±0.74
	21-25 day	18.32±0.57	30.41±1.05	18.27±0.48	30.27±0.58	18.36±0.44	30.21±0.42	18.41±1.29	29.84±1.02
	26-30 day	21.90±0.43	29.99±0.73	21.90±0.40	29.97±0.49	21.89±0.54	29.87±0.71	21.94±0.80	29.55±0.99
	31-35 day	23.24±0.61	34.74±0.71	23.17±0.75	34.72±0.60	23.27±0.60	34.76±0.62	23.30±0.74	34.31±0.94
	36-40 day	26.26±0.98	38.95±1.06	26.08±0.74	38.75±0.77	26.34±0.64	39.00±0.84	26.27±0.94	38.68±1.21
	41-45 day	27.41±0.63	41.56±1.25	27.28±0.67	41.52±0.92	27.42±0.58	41.87±0.97	27.53±0.71	41.43±1.25
	46-50 day	28.35±0.67	42.53±1.06	28.33±0.82	42.24±0.88	28.47±0.81	42.71±1.00	28.42±0.69	42.66±1.10
	51-55 day	28.54±0.56	44.23±1.02	28.28±0.52	43.86±0.79	28.52±0.40	44.45±1.03	28.59±0.38	44.44±0.87
	56-60 day	29.68±0.75	44.95±0.94	29.51±0.78	44.55±0.89	29.73±0.62	45.12±0.83	29.73±0.78	45.29±0.66
Average daily food intake of rats (g/day/rat)		21.05±0.61	30.59±0.79	21.01±0.61	30.27±1.03	21.10±0.55	30.65±0.64	21.13±0.74	30.49±0.80

Note: The data were compared for statistical significance with the same-sex control group. In the same row, values with different small letter superscripts mean significant difference ($P<0.05$), while with the same or no letter superscripts mean no significant difference ($P>0.05$). The same as below. CON group, basal diet; Low group, basal diet + 2000 mg/kg; Middle group, basal diet + 10000 mg/kg. High group, basal diet + 50000 mg/kg.

Table S2-2. Effect of Sublancin on the daily food intake of SD rats fed for 120 days during chronic toxicity trial.

Groups		CON		Low		Middle		High	
Experimental rats		♀ 20	♂ 20	♀ 20	♂ 20	♀ 20	♂ 20	♀ 20	♂ 20
Average food intake of rats during each experimental period (g/day/rat)	61-65 day	28.18±0.80	46.16±0.82	27.65±0.58	46.09±0.95	28.02±0.65	46.44±0.81	28.34±0.92	46.58±0.92
	66-70 day	27.87±0.70	47.66±0.69	28.18±2.42	47.56±0.62	27.73±0.62	48.02±0.99	27.79±1.12	48.19±0.85
	71-75 day	27.07±0.68	46.11±0.71	27.38±2.17	46.07±0.76	26.82±0.65	46.69±1.01	27.34±1.57	46.56±0.53
	76-80 day	26.00±0.61	44.26±0.62	26.61±2.71	44.38±0.75	25.67±0.85	44.84±0.99	25.77±1.26	44.60±0.81
	81-85 day	25.51±0.61	42.91±0.60	26.22±2.77	43.12±0.74	25.26±0.78	43.57±0.87	25.26±1.36	43.38±1.02
	86-90 day	25.26±0.63	41.80±0.67	25.91±2.67	41.99±0.80	24.91±0.89	42.47±0.77	24.97±1.40	41.79±0.55
	91-95 day	25.23±0.67	41.23±0.72	25.73±2.20	41.46±0.95	24.88±0.85	41.97±1.05	24.82±1.58	41.56±0.82
	96-100 day	24.86±0.59	40.84±0.71	25.30±1.99	41.10±1.00	24.46±0.74	41.47±1.06	24.52±1.54	41.17±0.82
	101-105 day	23.84±0.52	39.89±0.69	24.15±1.84	40.17±0.88	23.52±0.76	40.45±1.34	23.45±1.38	40.16±0.87
	106-110 day	22.70±0.55	38.40±0.68	23.24±2.11	38.62±0.84	22.36±0.59	38.95±1.13	22.41±1.44	38.69±0.82
	111-115 day	22.28±0.56	37.81±0.53	23.00±2.30	38.03±0.64	21.86±0.61	38.38±1.38	21.94±1.39	38.20±0.79
	116-120 day	21.53±0.54	36.90±0.60	22.42±2.76	37.14±0.53	21.22±0.67	37.53±1.27	21.20±1.40	37.24±0.85
Average daily food intake of rats (g/day/rat)		25.03±0.62	42.00±0.67	25.48±2.21	42.14±0.79	24.73±0.72	42.56±1.05	24.82±1.36	42.34±0.80

Note: The data were compared for statistical significance with the same-sex control group. In the same row, values with different small letter superscripts mean significant difference (P<0.05), while with the same or no letter superscripts mean no significant difference (P>0.05). The same as below. CON group, basal diet; Low group, basal diet + 2000 mg/kg; Middle group, basal diet + 10000 mg/kg. High group, basal diet + 50000 mg/kg.

Table S2-3. Effect of Sublancin on the daily food intake of SD rats fed for 180 days during chronic toxicity trial.

Groups		CON		Low		Middle		High	
Experimental rats		♀ 20	♂ 20	♀ 20	♂ 20	♀ 20	♂ 20	♀ 20	♂ 20
Average food intake of rats during each experimental period (g/day/rat)	121-125 day	20.79±0.84	37.44±0.68	21.60±1.68	37.64±0.44	20.70±0.54	38.08±1.06	20.29±1.47	37.48±1.13
	126-130 day	20.28±0.80	37.18±0.72	21.23±1.97	37.39±0.66	20.28±0.45	37.78±0.99	19.84±1.39	36.98±1.01
	131-135 day	20.14±0.52	35.78±0.79	21.04±2.05	35.97±0.85	19.87±0.45	36.25±1.08	19.57±1.32	35.72±1.07
	136-140 day	19.62±0.71	34.99±0.61	20.61±2.28	35.10±0.88	19.34±0.88	35.42±1.18	19.28±1.38	34.82±1.19
	141-145 day	19.18±0.68	35.19±0.56	20.07±1.96	35.23±0.90	19.21±0.62	35.63±0.96	18.75±1.17	35.02±1.34
	146-150 day	18.60±0.42	35.41±0.61	19.56±2.28	35.43±0.76	18.67±0.52	35.88±1.06	18.15±1.30	35.16±1.17
	151-155 day	18.75±0.58	35.30±0.64	19.37±1.70	35.34±0.87	18.75±0.50	35.78±1.04	18.26±1.41	35.02±1.21
	156-160 day	18.81±0.80	35.49±0.60	19.57±1.97	35.54±0.92	18.75±0.62	36.01±0.96	18.36±1.27	35.15±1.50
	161-165 day	18.97±0.82	34.81±0.85	19.45±1.32	34.92±0.82	18.92±0.66	35.36±1.03	18.53±1.18	34.39±1.28
	166-170 day	19.05±0.64	33.55±0.77	19.44±1.23	33.60±0.88	18.96±0.72	34.09±1.02	18.70±1.29	33.21±0.90
	171-175 day	19.10±0.64	33.67±0.77	19.31±1.17	33.74±0.82	18.98±0.74	34.24±1.06	18.71±1.16	33.43±1.06
	176-180 day	19.16±0.62	33.74±0.79	19.12±0.78	33.81±0.79	19.01±0.87	34.31±1.13	18.85±1.09	30.45±1.40
Average daily food intake of rats (g/day/rat)		19.37±0.67	35.21±0.70	20.03±1.70	35.31±0.80	19.29±0.63	35.74±1.05	18.94±1.28	34.74±1.19

Note: The data were compared for statistical significance with the same-sex control group. In the same row, values with different small letter superscripts mean significant difference ($P<0.05$), while with the same or no letter superscripts mean no significant difference ($P>0.05$). The same as below. CON group, basal diet; Low group, basal diet + 2000 mg/kg; Middle group, basal diet + 10000 mg/kg. High group, basal diet + 50000 mg/kg.

Table S3. Effect of sublancin on the daily water intake of SD rats fed for 45 and 60 days during subchronic toxicity trial.

Groups		CON		Low		Middle		High	
Experimental rats		♀ 20	♂ 20	♀ 20	♂ 20	♀ 20	♂ 20	♀ 20	♂ 20
Average water intake of rats during each experimental period (g/day/rat)	1-5 day	13.80±0.96	14.82±0.63	12.73±0.60	14.80±0.85	12.73±0.60	14.80±0.85	13.66±0.35	14.34±0.94
	6-10 day	20.79±1.10	23.35±0.91	20.08±0.92	23.27±1.42	20.08±0.92	23.27±1.42	20.66±0.73	22.92±1.48
	11-15 day	28.91±1.32	36.95±2.23	28.00±0.85	36.30±1.99	28.00±0.85	36.30±1.99	28.91±0.70	35.55±2.49
	16-20 day	35.56±0.38	46.67±1.99	35.20±1.80	48.36±4.20	35.20±1.80	48.36±4.20	36.02±1.10	46.31±2.14
	21-25 day	39.47±1.02	53.80±2.28	40.09±4.41	55.57±3.31	40.09±4.41	55.57±3.31	39.05±1.11	53.93±1.73
	26-30 day	42.91±0.16	62.94±3.17	42.54±1.03	62.25±2.26	42.54±1.03	62.25±2.26	41.85±1.84	60.47±1.98
	31-35 day	44.11±0.75	67.58±2.07	45.02±2.77	65.96±4.38	45.02±2.77	65.96±4.38	40.84±2.25	67.88±2.27
	36-40 day	47.91±1.82	74.24±0.91	43.47±3.62	73.83±3.10	43.47±3.62	73.83±3.10	44.20±2.63	72.77±0.91
	41-45 day	47.94±4.73	78.91±1.16	44.08±4.13	76.86±5.90	44.08±4.13	76.86±5.90	48.12±0.89	77.56±1.63
Average daily water intake of rats (g/day/rat)		35.71±1.36	51.03±1.17	34.58±2.24	50.80±5.90	34.58±2.24	50.80±3.05	34.81±1.29	50.28±1.85
Average water intake of rats during each experimental period (g/day/rat)	46-50 day	49.79±1.56	79.51±2.25	52.56±7.18	78.88±0.52	49.54±2.23	80.38±2.64	49.71±0.83	78.06±2.62
	51-55 day	49.83±1.55	79.48±2.24	52.57±7.14	78.87±0.53	49.52±2.18	79.62±2.78	49.70±0.89	78.07±2.62
	56-60 day	46.59±1.38	81.88±3.23	47.03±2.21	81.93±0.87	46.93±1.12	82.50±1.98	48.57±3.34	80.31±2.41
	61-65 day	47.28±0.94	84.58±4.04	46.03±1.02	83.48±1.63	47.61±0.74	82.39±6.79	47.62±0.86	82.87±1.82
	66-70 day	43.71±1.90	84.24±0.95	44.42±3.25	83.22±3.79	45.07±0.94	85.38±7.23	48.28±0.50	82.66±2.56
	71-75 day	43.45±3.35	83.73±4.35	44.17±0.67	85.74±4.10	45.81±0.95	81.38±6.96	47.65±2.41	81.91±1.93
	76-80 day	44.47±2.61	83.10±0.52	42.16±0.42	78.11±1.52	46.38±0.67	82.55±7.56	46.46±0.80	84.58±2.85
	81-85 day	42.65±1.16	76.49±3.70	41.43±0.00	75.92±2.77	43.01±1.30	80.40±5.64	45.44±3.14	87.08±2.27
	86-90 day	42.89±3.08	78.68±3.84	40.43±0.17	77.73±3.05	42.60±2.20	79.21±0.24	43.35±2.24	82.70±2.48
Average daily water intake of rats (g/day/rat)		45.63±1.95	81.30±2.79	45.64±2.45	80.43±2.09	46.27±1.37	81.53±4.65	47.42±1.67	82.03±2.40

Note: The data were compared for statistical significance with the same-sex control group. In the same row, values with different small letter superscripts mean significant difference ($P<0.05$), while with the same or no letter superscripts mean no significant difference ($P>0.05$). The same as below. CON group, basal diet; Low group, basal diet + 2000 mg/kg; Middle group, basal diet + 10000 mg/kg. High group, basal diet + 50000 mg/kg.

Table S4-1. Effect of sublancin on the daily water intake of SD rats fed for 60 days during chronic toxicity trial.

Groups		CON		Low		Middle		High	
Experimental rats		♀ 20	♂ 20	♀ 20	♂ 20	♀ 20	♂ 20	♀ 20	♂ 20
Average water intake of rats during each experimental period (g/day/rat)	1-5 day	14.01±0.53	16.32±0.38	14.04±0.45	16.40±0.35	14.11±0.84	16.39±0.28	14.16±0.62	16.36±0.31
	6-10 day	19.93±0.63	24.26±0.69	20.05±0.54	24.72±0.70	19.98±0.91	24.48±0.50	19.99±0.55	24.46±0.34
	11-15 day	27.41±1.43	37.20±1.25	27.49±1.27	37.49±0.68	27.56±1.59	37.07±0.79	27.51±1.29	37.07±0.95
	16-20 day	36.03±1.72	50.51±1.36	35.79±1.46	50.92±0.72	36.31±1.56	50.42±1.30	36.52±2.89	50.28±1.74
	21-25 day	41.58±1.29	58.45±2.01	41.38±0.95	58.70±0.97	41.98±1.65	58.07±0.81	41.80±2.92	57.36±1.96
	26-30 day	41.42±0.80	63.10±1.55	41.16±0.64	63.61±0.46	41.59±1.43	62.85±1.49	41.50±1.52	62.18±2.09
	31-35 day	39.32±1.04	65.47±1.33	38.93±1.00	66.03±0.84	39.47±1.35	65.51±1.17	39.42±1.25	64.65±1.78
	36-40 day	41.44±1.55	69.83±1.90	41.07±1.06	70.25±1.15	41.45±1.48	69.92±1.50	41.45±1.48	69.35±2.16
	41-45 day	40.88±0.94	70.85±2.12	40.59±0.92	71.46±1.24	41.07±1.11	71.38±1.65	41.06±1.05	70.63±2.13
	46-50 day	42.46±1.00	75.05±1.86	42.04±0.96	75.30±0.88	42.62±1.15	75.37±1.76	42.56±1.03	75.29±1.94
	51-55 day	43.83±0.86	78.74±1.82	43.21±0.72	78.76±1.31	44.01±0.81	79.13±1.83	43.92±0.58	79.11±1.56
	56-60 day	44.68±1.13	84.38±1.76	44.10±0.98	84.43±1.26	44.83±1.18	84.70±1.55	44.76±1.18	85.03±1.24
Average daily water intake of rats (g/day/rat)		36.08±1.08	57.85±1.50	35.82±0.91	58.17±0.88	36.25±1.25	57.94±1.22	36.22±1.36	57.65±1.52

Note: The data were compared for statistical significance with the same-sex control group. In the same row, values with different small letter superscripts mean significant difference ($P<0.05$), while with the same or no letter superscripts mean no significant difference ($P>0.05$). The same as below. CON group, basal diet; Low group, basal diet + 2000 mg/kg; Middle group, basal diet + 10000 mg/kg. High group, basal diet + 50000 mg/kg.

Table S4-2. Effect of sublancin on the daily water intake of SD rats fed for 120 days during chronic toxicity trial.

**Groups**		**CON**		**Low**		**Middle**		**High**	
**Experimental rats**		♀<br>**20**	♂<br>**20**	♀<br>**20**	♂<br>**20**	♀<br>**20**	♂<br>**20**	♀<br>**20**	♂<br>**20**
Average water intake of rats during each experimental period (g/day/rat)	61-65 day	44.95±1.28	83.85±1.05	43.97±0.81	83.17±0.87	45.06±1.58	82.88±1.45	45.24±1.49	83.13±1.64
	66-70 day	44.11±1.11	82.38±1.46	43.01±0.77	81.45±0.54	44.12±1.27	81.66±1.68	44.20±1.55	81.90±1.43
	71-75 day	42.35±1.05	81.04±1.17	41.33±0.90	80.96±1.29	42.24±1.20	81.19±1.75	42.25±1.78	81.06±0.91
	76-80 day	40.47±0.95	80.29±1.12	39.48±0.93	79.79±0.55	40.30±1.49	79.98±1.76	40.16±1.91	79.55±1.44
	81-85 day	39.33±0.95	78.94±1.11	38.45±0.97	78.38±0.94	39.09±1.20	78.47±1.56	38.94±2.10	78.12±1.83
	86-90 day	38.69±0.96	77.29±1.09	37.81±0.96	77.16±1.04	38.36±1.43	77.24±1.40	38.26±2.14	76.81±1.75
	91-95 day	36.83±0.98	76.02±1.22	36.08±0.83	76.22±1.26	36.35±1.34	76.27±1.90	36.33±2.24	75.53±1.49
	96-100 day	36.62±0.87	74.94±1.31	35.93±0.55	74.18±1.17	36.08±1.20	73.86±1.89	36.12±2.27	73.44±1.47
	101-105 day	35.22±0.78	72.75±1.26	34.53±0.78	72.73±1.19	34.64±1.19	72.36±2.39	34.64±2.04	71.99±1.55
	106-110 day	33.97±0.82	71.37±1.24	33.29±0.68	70.16±1.12	33.49±1.07	69.96±2.02	33.54±2.16	69.59±1.31
	111-115 day	32.76±0.82	68.98±1.22	32.13±0.61	68.93±1.12	32.34±1.15	68.99±2.48	32.25±2.05	68.67±1.42
	116-120 day	32.05±0.80	67.98±0.95	31.46±0.82	66.81±0.94	31.69±1.13	67.00±2.27	31.55±2.08	66.49±1.52
Average daily water intake of rats (g/day/rat)		38.11±0.95	76.32±1.08	37.29±0.80	75.83±1.00	37.81±1.27	75.82±1.88	37.79±1.98	75.52±1.50

Note: The data were compared for statistical significance with the same-sex control group. In the same row, values with different small letter superscripts mean significant difference (P<0.05), while with the same or no letter superscripts mean no significant difference (P>0.05). The same as below. CON group, basal diet; Low group, basal diet + 2000 mg/kg; Middle group, basal diet + 10000 mg/kg. High group, basal diet + 50000 mg/kg.

Table S4-3. Effect of sublancin on the daily water intake of SD rats fed for 180 days during chronic toxicity trial.

Groups		CON		Low		Middle		High	
Experimental rats		♀ 20	♂ 20	♀ 20	♂ 20	♀ 20	♂ 20	♀ 20	♂ 20
Average water intake of rats during each experimental period (g/day/rat)	121-125 day	31.91±1.28	65.82±1.18	32.00±0.60	65.36±0.81	31.91±0.92	66.04±1.84	31.14±2.25	65.01±1.97
	126-130 day	30.72±1.21	64.94±1.18	30.86±0.79	64.26±1.13	30.70±0.69	64.93±1.71	30.05±2.11	63.87±2.17
	131-135 day	30.99±0.80	63.91±1.24	31.19±1.01	64.56±1.61	30.93±0.87	64.80±1.93	30.32±2.04	63.74±1.95
	136-140 day	30.63±1.10	63.96±1.40	30.82±1.22	63.99±1.70	30.68±1.06	64.31±2.13	30.10±2.15	63.22±2.16
	141-145 day	29.80±1.06	63.53±1.11	29.98±1.12	61.98±1.72	29.94±0.83	62.48±1.69	29.12±1.82	61.41±2.34
	146-150 day	29.68±0.67	61.71±0.99	29.87±1.00	61.69±1.42	29.87±0.81	62.28±1.84	28.96±2.07	61.12±1.90
	151-155 day	29.62±0.91	61.46±1.05	29.72±1.01	60.29±1.59	29.67±0.75	60.84±1.77	28.84±2.23	59.56±2.06
	156-160 day	29.72±1.26	60.03±1.08	29.83±1.04	59.55±1.70	29.79±0.83	60.10±1.60	29.00±2.00	58.66±2.51
	161-165 day	29.55±1.28	59.24±1.00	29.61±1.03	58.93±1.54	29.60±0.87	59.41±1.73	28.88±1.84	58.02±2.30
	166-170 day	29.06±0.98	58.49±1.42	29.06±1.16	57.25±1.66	29.06±0.82	57.77±1.41	28.53±1.97	57.21±2.33
	171-175 day	29.12±0.98	56.85±1.30	29.02±1.21	55.59±1.48	28.98±0.84	56.13±1.25	28.53±1.76	55.86±2.13
	176-180 day	28.70±0.93	55.20±1.26	28.52±1.40	54.50±1.35	28.49±0.83	55.19±1.83	28.23±1.63	54.99±1.90
Average daily water intake of rats (g/day/rat)		29.96±1.04	61.26±1.27	30.04±1.05	60.66±1.48	29.72±4.64	61.19±1.69	29.31±1.99	59.72±4.64

Note: The data were compared for statistical significance with the same-sex control group. In the same row, values with different small letter superscripts mean significant difference (P<0.05), while with the same or no letter superscripts mean no significant difference (P>0.05). The same as below. CON group, basal diet; Low group, basal diet + 2000 mg/kg; Middle group, basal diet + 10000 mg/kg. High group, basal diet + 50000 mg/kg.

Table S5. Effect of sublancin on the average daily gain of SD rats fed for 45 and 90 days during subchronic toxicity trial.

Groups		CON		Low		Middle		High	
Experimental rats		♀ 10	♂ 20	♀ 20	♂ 20	♀ 20	♂ 20	♀ 20	♂ 20
Average daily gain of rats during each experimental period (g/day/rat)	1-5 day	4.14±1.49	4.16±1.31	3.98±0.80	4.68±0.94	3.70±0.74	4.70±0.94	4.52±0.90	4.58±0.92
	6-10 day	5.86±1.62	7.30±1.35	5.94±1.19	7.62±1.52	6.60±1.32	7.00±1.40	5.86±1.17	7.36±1.47
	11-15 day	4.70±1.57	8.62±2.49	4.08±0.82	8.56±1.71	4.48±0.90	8.38±1.68	5.02±1.00	8.52±1.70
	16-20 day	3.74±1.56	7.72±2.40	3.60±0.72	7.46±1.49	3.54±0.71	7.92±1.58	3.32±0.66	7.52±1.50
	21-25 day	3.04±1.17	6.66±1.66	2.78±0.56	6.62±1.32	2.74±0.55	6.52±1.30	2.66±0.53	6.46±1.29
	26-30 day	2.76±1.49	6.38±0.93	2.92±0.58	6.84±1.37	3.38±0.68	6.16±1.23	2.78±0.56	5.70±1.14
	31-35 day	1.86±0.77	4.58±1.97	2.24±0.45	4.50±0.90	1.90±0.38	4.92±0.98	2.16±0.43	5.56±1.11
	36-40 day	1.74±1.03	4.40±1.43	1.76±0.35	4.00±0.80	1.58±0.32	4.38±0.88	1.74±0.35	3.54±0.71
	41-45 day	1.86±0.77	3.82±0.58	2.08±0.42	4.04±0.81	1.66±0.33	4.04±0.81	1.98±0.40	3.78±0.76
Total average daily gain of rats (g/day/rat)		3.30±1.28	5.96±1.57	3.26±0.65	6.04±1.21	3.29±0.66	6.00±1.20	3.34±0.67	5.89±1.18
Average daily gain of rats during each experimental period (g/day/rat)	46-50 day	0.64±0.60	2.92±0.36	1.00±0.20	2.92±0.58	0.68±0.14	3.28±0.49	0.92±0.18	3.14±0.69
	51-55 day	0.84±0.71	3.08±0.90	0.64±0.13	3.20±0.64	1.28±0.26	2.88±0.58	0.68±0.14	2.84±0.57
	56-60 day	0.92±0.36	2.88±0.78	0.88±0.18	2.40±0.48	0.72±0.14	3.04±0.61	0.96±0.19	2.68±0.54
	61-65 day	0.56±0.68	2.88±0.46	0.72±0.14	2.72±0.54	0.76±0.15	2.92±0.58	0.60±0.12	2.88±0.58
	66-70 day	0.80±0.34	2.80±1.36	0.76±0.15	2.36±0.47	0.42±0.18	2.68±0.54	1.04±0.21	2.48±0.50
	71-75 day	0.32±0.58	2.68±0.41	0.28±0.06	2.20±0.44	0.56±0.11	2.52±0.50	0.72±0.14	2.52±0.50
	76-80 day	0.68±0.53	1.72±1.03	0.88±0.18	2.28±0.46	0.64±0.13	2.31±0.47	0.62±0.11	2.60±0.52
	81-85 day	0.40±0.65	1.88±0.33	0.32±0.06	2.04±0.41	0.84±0.17	2.20±0.44	0.32±0.06	1.80±0.36
	86-90 day	1.00±0.84	1.96±1.04	0.68±0.14	1.80±0.36	0.76±0.15	1.92±0.38	0.80±0.16	1.52±0.30
Total average daily gain of rats (g/day/rat)		0.68±0.57	2.53±0.74	0.68±0.14	2.44±0.49	0.74±0.16	2.58±0.52	0.74±0.15	2.46±0.49

Note: The data were compared for statistical significance with the same-sex control group. In the same row, values with different small letter superscripts mean significant difference ($P<0.05$), while with the same or no letter superscripts mean no significant difference ($P>0.05$). The same as below. CON group, basal diet; Low group, basal diet + 2000 mg/kg; Middle group, basal diet + 10000 mg/kg. High group, basal diet + 50000 mg/kg.

Table S6-1. Effect of sublancin on the average daily gain of SD rats fed for 60 days during chronic toxicity trial.

**Groups**		**CON**		**Low**		**Middle**		**High**	
**Experimental rats**		♀ 20	♂ 20	♀ 20	♂ 20	♀ 20	♂ 20	♀ 20	♂ 20
Average daily gain of rats during each experimental period (g/day/rat)	1-5 day	4.08±0.47	4.94±0.63	3.86±0.73	4.91±0.65	4.09±1.38	4.99±0.70	4.08±0.78	4.97±0.59
	6-10 day	4.86±1.20	5.89±1.18	4.96±1.37	6.07±0.90	4.78±2.63	6.05±1.09	4.73±1.59	6.06±0.81
	11-15 day	4.26±2.23	7.27±1.36	4.20±2.31	7.16±0.98	4.36±2.42	6.9±1.20	4.29±1.65	6.92±1.30
	16-20 day	3.86±1.85	7.23±1.55	3.55±2.18	7.27±0.94	3.95±1.90	7.27±1.07	4.19±2.15	7.16±2.52
	21-25 day	3.38±2.68	6.79±1.82	3.43±1.90	6.49±1.14	3.48±2.28	6.56±1.04	3.13±2.40	6.08±1.22
	26-30 day	2.98±2.34	5.75±1.84	2.91±1.58	5.94±1.04	2.79±1.75	5.85±1.16	2.87±2.26	5.86±1.14
	31-35 day	2.32±2.12	5.43±1.21	2.14±1.41	5.41±0.90	2.31±1.46	5.67±0.95	2.34±1.82	5.47±1.47
	36-40 day	2.18±1.87	4.94±1.50	2.22±1.73	4.66±1.17	2.05±2.08	5.00±1.01	2.01±2.34	5.25±1.62
	41-45 day	1.80±1.36	4.35±2.10	1.86±1.38	4.61±1.76	1.99±1.73	4.78±1.24	1.98±1.80	4.58±1.50
	46-50 day	1.75±1.67	4.10±2.66	1.61±1.28	3.66±1.43	1.72±1.44	3.89±1.64	1.67±1.45	4.54±1.80
	51-55 day	1.58±1.46	3.60±2.19	1.35±1.53	3.46±1.23	1.59±1.74	3.67±1.45	1.55±1.54	3.73±1.72
	56-60 day	1.50±1.41	3.40±1.50	1.53±0.88	3.32±1.56	1.48±0.75	3.32±1.42	1.50±1.67	3.65±1.73
Total average daily gain of rats (g/day/rat)		2.88±1.72	5.31±1.63	2.80±1.52	5.25±1.14	2.88±1.80	5.33±1.16	2.87±1.79	5.36±1.45

Note: The data were compared for statistical significance with the same-sex control group. In the same row, values with different small letter superscripts mean significant difference (P<0.05), while with the same or no letter superscripts mean no significant difference (P>0.05). The same as below. CON group, basal diet; Low group, basal diet + 2000 mg/kg; Middle group, basal diet + 10000 mg/kg. High group, basal diet + 50000 mg/kg.

Table S6-2. Effect of sublancin on the average daily gain of SD rats fed for 120 days during chronic toxicity trial.

Groups		CON		Low		Middle		High	
Experimental rats		♀ 20	♂ 20	♀ 20	♂ 20	♀ 20	♂ 20	♀ 20	♂ 20
Average daily gain of rats during each experimental period (g/day/rat)	61-65 day	1.17±0.90	3.37±0.77	1.03±0.90	3.20±1.16	1.08±0.70	3.36±1.31	1.35±0.66	3.44±1.17
	66-70 day	1.37±0.62	3.08±0.87	1.17±0.67	3.04±1.42	1.25±0.64	3.24±1.12	1.15±0.54	3.24±1.22
	71-75 day	1.09±0.69	2.97±0.84	1.11±0.57	3.16±1.26	0.95±0.46	3.32±1.31	0.87±0.67	2.92±1.20
	76-80 day	1.08±0.70	2.92±0.94	1.04±0.60	3.16±1.26	0.99±0.72	3.12±1.22	0.79±0.53	2.76±1.26
	81-85 day	0.71±0.60	2.60±1.46	0.80±0.49	2.80±1.26	0.60±0.69	2.84±1.32	0.57±0.72	2.92±1.20
	86-90 day	0.68±0.95	2.33±0.99	0.64±0.51	2.32±1.20	0.53±0.63	2.44±1.12	0.60±0.31	2.32±1.20
	91-95 day	0.59±0.95	2.43±1.02	0.72±0.74	2.52±1.34	0.33±0.54	2.64±1.24	0.44±0.65	2.20±1.16
	96-100 day	0.56±0.95	2.07±0.72	0.63±0.89	2.16±1.41	0.44±0.60	1.85±1.33	0.55±0.58	2.25±1.24
	101-105 day	0.51±0.54	1.93±1.19	0.47±0.66	2.04±1.21	0.41±0.61	1.83±1.43	0.35±0.72	1.87±1.57
	106-110 day	0.45±0.60	1.93±1.27	0.40±0.82	1.80±1.21	0.57±0.62	1.99±1.41	0.65±0.89	1.97±1.15
	111-115 day	0.61±0.69	1.83±1.12	0.67±0.71	1.84±1.12	0.69±0.58	1.93±1.41	0.45±0.80	1.97±1.08
	116-120 day	0.55±0.82	1.69±0.93	0.57±0.76	1.79±0.96	0.64±0.54	1.93±1.22	0.53±0.51	1.61±1.23
Total average daily gain of rats (g/day/rat)		0.78±0.75	2.43±1.01	0.77±0.69	2.49±1.23	0.71±0.61	2.45±1.29	0.69±0.63	2.46±1.22

Note: The data were compared for statistical significance with the same-sex control group. In the same row, values with different small letter superscripts mean significant difference ($P<0.05$), while with the same or no letter superscripts mean no significant difference ($P>0.05$). The same as below. CON group, basal diet; Low group, basal diet + 2000 mg/kg; Middle group, basal diet + 10000 mg/kg. High group, basal diet + 50000 mg/kg.

Table S6-3. Effect of sublancin on the average daily gain of SD rats fed for 180 days during chronic toxicity trial, g/day/rat.

Groups		CON		Low		Middle		High	
Experimental rats		♀ 20	♂ 20	♀ 20	♂ 20	♀ 20	♂ 20	♀ 20	♂ 20
Average daily gain of rats during each experimental period (g/day/rat)	121-125 day	0.60±1.29	1.48±1.13	0.66±0.84	1.50±1.21	0.60±0.51	1.44±0.65	0.84±0.85	1.68±1.17
	126-130 day	0.52±0.74	1.14±0.69	0.62±0.76	1.20±0.90	0.48±0.64	1.04±1.05	0.64±0.74	0.96±1.29
	131-135 day	0.70±0.99	1.20±0.62	0.82±1.02	1.18±1.41	0.62±1.12	0.90±0.91	0.68±1.03	0.88±1.19
	136-140 day	0.48±0.96	1.18±0.79	0.46±1.17	0.90±0.75	0.70±0.97	1.10±0.99	0.74±1.06	1.00±1.30
	141-145 day	0.54±0.81	0.92±0.61	0.56±0.91	0.70±1.09	0.74±0.87	0.96±0.86	0.22±0.88	0.92±1.30
	146-150 day	0.40±1.06	0.70±0.60	0.42±0.82	0.64±0.91	0.50±0.84	0.80±0.72	0.28±0.78	0.62±1.47
	151-155 day	0.52±1.10	0.68±0.75	0.34±0.73	0.76±1.07	0.22±0.69	0.72±0.37	0.38±0.74	0.70±1.21
	156-160 day	0.20±0.87	0.66±0.52	0.22±0.70	0.68±1.05	0.26±0.62	0.76±0.82	0.34±0.86	0.42±1.22
	161-165 day	0.32±1.12	0.50±0.96	0.20±1.02	0.70±0.95	0.26±0.67	0.66±1.03	0.38±0.91	0.70±1.71
	166-170 day	0.30±0.72	0.68±0.85	0.16±1.01	0.50±0.88	0.18±0.68	0.76±0.75	0.58±0.62	0.50±1.68
	171-175 day	0.38±0.49	0.44±1.13	0.18±0.82	0.52±0.47	0.10±0.52	0.52±0.57	0.26±0.75	0.74±1.56
	176-180 day	0.32±0.84	0.48±0.77	0.14±0.79	0.46±1.02	0.16±0.46	0.48±0.80	0.56±0.85	0.56±0.68
Total average daily gain of rats (g/day/rat)		0.44±0.92	0.84±0.79	0.40±0.88	0.81±0.98	0.40±0.72	0.84±0.79	0.49±0.84	0.81±1.31

Note: The data were compared for statistical significance with the same-sex control group. In the same row, values with different small letter superscripts mean significant difference (P<0.05), while with the same or no letter superscripts mean no significant difference (P>0.05). The same as below. CON group, basal diet; Low group, basal diet + 2000 mg/kg; Middle group, basal diet + 10000 mg/kg. High group, basal diet + 50000 mg/kg.

Table S7. Effect of sublancin on the hematology of SD rats fed for 45 and 90 days during subchronic toxicity trial.

Groups	Items	HGB（g/L）		RBC (10^{12}/L)		WBC (10^9/L)		PLT (10^9/L)		HCT (%)		EOS (10^9/L)		BAS (10^8/L)		MO (10^9/L)		LY (10^9/L)	
		♀	♂	♀	♂	♀	♂	♀	♂	♀	♂	♀	♂	♀	♂	♀	♂	♀	♂
		5	5	5	5	5	5	5	5	5	5	5	5	5	5	5	5	5	5
45 day	CON	156.58 ±10.57	148.88 ±10.04	7.10 ±0.45	6.89 ±0.59	8.47 ±1.61	8.86 ±1.08	475.42 ±68.98	465.37 ±70.75	35.42 ±4.54	41.74 ±7.24	0.25 ±0.18	0.38 ±0.29	0.85 ±0.67	0.91 ±0.86	0.21 ±0.09	0.24 ±0.08	4.09 ±1.01	5.93 ±1.76
	Low	160.13 ±12.68	153.15 ±5.57	7.37 ±0.58	7.03 ±0.25	8.35 ±1.48	9.00 ±0.72	441.44 ±56.96	469.62 ±57.63	35.22 ±5.28	38.41 ±5.94	0.24 ±0.17	0.48 ±0.31	1.17 ±0.74	1.32 ±1.15	0.21 ±0.10	0.28 ±0.11	4.51 ±1.72	5.11 ±1.47
	Middle	157.56 ±6.31	153.09 ±8.95	7.40 ±0.69	7.16 ±0.23	8.21 ±1.11	9.49 ±1.20	454.79 ±53.35	446.4 ±47.57	37.55 ±4.90	38.40 ±7.99	0.34 ±0.19	0.26 ±0.18	0.91 ±0.86	1.68 ±0.78	0.20 ±0.10	0.25 ±0.12	4.22 ±1.14	5.47 ±1.66
	High	151.60 ±8.48	155.76 ±8.66	6.93 ±0.57	7.27 ±0.52	8.03 ±0.93	9.32 ±0.64	480.90 ±69.92	491.69 ±74.40	35.03 ±5.48	38.39 ±7.99	0.31 ±0.17	0.26 ±0.19	0.77 ±1.03	1.66 ±0.79	0.19 ±0.10	0.25 ±0.12	4.25 ±1.18	5.02 ±1.93
90 day	CON	147.57 ±8.01	154.79 ±11.13	7.08 ±0.55	7.41 ±0.49	8.27 ±1.34	8.93 ±0.71	373.09 ±71.68	376.67 ±68.49	37.50 ±3.14	37.52 ±7.51	0.32 ±0.18	0.28 ±0.19	1.47 ±0.79	1.62 ±1.26	0.26 ±0.08	0.25 ±0.16	4.86 ±0.89	5.08 ±1.53
	Low	153.57 ±8.18	153.16 ±10.78	7.19 ±0.51	7.18 ±0.56	8.05 ±1.12	8.68 ±0.68	404.90 ±54.08	418.00 ±60.74	37.54 ±3,54	36.71 ±8.72	0.37 ±0.13	0.44 ±0.35	1.04 ±0.87	1.30 ±0.86	0.22 ±0.10	0.24 ±0.09	4.85 ±1.11	4.85 ±1.11
	Middle	150.09 ±11.09	153.58 ±11.60	7.26 ±0.46	7.60 ±0.53	8.07 ±1.18	9.22 ±0.96	374.70 ±60.49	401.67 ±67.92	35.88 ±5.04	36.71 ±6.37	0.27 ±0.19	0.38 ±0.23	1.45 ±0.66	1.45 ±1.02	0.24 ±0.08	0.27 ±0.10	4.24 ±1.24	4.69 ±1.10
	High	147.38 ±7.07	151.77 ±9.95	7.47 ±0.84	7.70 ±0.34	7.87 ±0.79	8.83 ±0.76	388.68 ±64.25	405.37 ±60.43	36.70 ±4.27	40.07 ±6.38	0.24 ±0.20	0.30 ±0.15	1.16 ±0.73	1.17 ±1.35	0.24 ±0.07	0.26 ±0.18	4.48 ±1.32	4.87 ±1.25

Note: The data were compared for statistical significance with the same-sex control group. In the same row, values with different small letter superscripts mean significant difference ($P<0.05$), while with the same or no letter superscripts mean no significant difference ($P>0.05$). The same as below. CON group, basal diet; Low group, basal diet + 2000 mg/kg; Middle group, basal diet + 10000 mg/kg. High group, basal diet + 50000 mg/kg. HGB =hemoglobin; RBC =red blood cell count; WBC =white blood cell count; PLT= platelet; HCT= hematocrit; EOS= eosinophils; BAS =basophils; MO =monocytes; and LY=lymphocytes.

Table S8. Effect of sublancin on the hematology of SD rats fed for 60, 120 and 180 days during chronic toxicity trial.

Groups \ Items		HGB（g/L）		RBC (10^{12}/L)		WBC (10^9/L)		PLT (10^9/L)		HCT (%)		EOS (10^9/L)		BAS (10^8/L)		MO (10^9/L)		LY (10^9/L)	
		♀	♂	♀	♂	♀	♂	♀	♂	♀	♂	♀	♂	♀	♂	♀	♂	♀	♂
		5	5	5	5	5	5	5	5	5	5	5	5	5	5	5	5	5	5
60 day	CON	148.72 ±17.56	150.17 ±15.47	8.22 ±0.62	7.84 ±1.13	7.56 ±1.43	6.94 ±0.91	562.63 ±36.88	554.83 ±29.87	40.30 ±1.50	39.70 ±2.85	0.01 ±0.00	0.01 ±0.00	0.01 ±0.00	0.01 ±0.00	0.27 ±0.13	0.32 ±0.10	4.60 ±1.05	4.65 ±0.98
	Low	150.67 ±15.23	151.26 ±12.74	7.80 ±0.38	8.16 ±0.66	7.50 ±1.05	7.28 ±0.58	561.65 ±34.86	582.06 ±36.12	39.50 ±3.50	38.80 ±2.42	0.01 ±0.00	0.01 ±0.00	0.01 ±0.00	0.01 ±0.00	0.24 ±0.13	0.30 ±0.09	4.21 ±1.25	4.00 ±1.19
	Middle	148.72 ±9.62	150.95 ±13.97	8.03 ±0.48	8.06 ±0.90	7.28 ±1.07	7.16 ±0.47	574.04 ±74.07	583.01 ±70.06	39.30 ±2.90	38.58 ±3.54	0.01 ±0.00	0.01 ±0.00	0.01 ±0.00	0.01 ±0.00	0.32 ±0.13	0.30 ±0.19	4.40 ±1.24	4.65 ±1.37
	High	150.94 ±8.56	144.24 ±7.60	8.04 ±0.62	8.22 ±0.44	6.82 ±0.94	7.04± 0.67	593.29 ±57.97	566.69 ±46.60	37.00 ±5.00	38.66 ±3.74	0.01 ±0.00	0.01 ±0.00	0.01 ±0.00	0.01 ±0.00	0.24 ±0.12	0.32 ±0.11	4.23 ±0.72	4.05 ±0.85
120 day	CON	145.78 ±4.94	147.87 ±11.43	8.31 ±0.55	8.52 ±1.04	7.06 ±0.85	7.60 ±1.32	582.17 ±67.90	551.97 ±59.47	39.70 ±2.30	39.50 ±2.20	0.01 ±0.00	0.01 ±0.00	0.01 ±0.00	0.01 ±0.00	0.28 ±0.08	0.29 ±0.18	4.25 ±1.85	4.42 ±1.78
	Low	144.41 ±12.42	152.50 ±9.51	8.64 ±0.74	8.55 ±1.06	6.88 ±1.81	7.04 ±1.51	574.55 ±64.23	580.66 ±18.00	36.40 ±4.10	37.50 ±3.50	0.01 ±0.00	0.01 ±0.00	0.01 ±0.00	0.01 ±0.00	0.24 ±0.09	0.31 ±0.11	4.48 ±1.47	4.62 ±1.36
	Middle	148.29 ±8.31	146.34 ±7.34	8.65 ±1.42	8.48 ±0.64	7.08 ±0.96	7.48 ±1.00	578.90 ±60.13	561.58 ±37.51	39.93 ±5.30	37.60 ±3.00	0.01 ±0.00	0.01 ±0.00	0.01 ±0.00	0.01 ±0.00	0.34 ±0.11	0.29 ±0.09	4.49 ±1.02	4.62 ±1.44
	High	152.32 ±11.57	143.68 ±12.85	8.21 ±0.51	8.41 ±0.66	7.14 ±1.31	7.38 ±1.30	567.44 ±44.54	536.09 ±50.08	37.00 ±3.60	37.50 ±3.60	0.01 ±0.00	0.01 ±0.00	0.01 ±0.00	0.01 ±0.00	0.26 ±0.11	0.29 ±0.14	4.26 ±1.17	5.07 ±0.89
180 day	CON	145.59 ±7.50	149.09 ±7.15	8.41 ±0.43	8.06 ±1.00	7.50 ±1.40	7.94 ±0.79	563.58 ±53.11	561.73 ±33.92	38.90 ±2.40	36.60 ±4.60	0.01 ±0.00	0.01 ±0.00	0.01 ±0.00	0.01 ±0.00	0.32 ±0.18	0.29 ±0.21	5.06 ±1.39	5.67 ±1.02
	Low	141.53 ±9.37	146.49 ±8.84	8.43 ±0.98	8.62 ±0.47	7.56 ±0.63	7.44 ±1.27	575.12 ±58.47	555.00 ±69.09	37.10 ±3.70	38.50 ±3.00	0.01 ±0.00	0.01 ±0.00	0.01 ±0.00	0.01 ±0.00	0.31 ±0.19	0.29 ±0.15	5.45 ±1.35	5.46 ±1.32
	Middle	147.27 ±4.90	145.29 ±6.35	8.22 ±0.87	8.31 ±0.76	7.98 ±0.61	7.50 ±1.09	585.85 ±37.72	573.94 ±60.61	39.40 ±2.20	38.50 ±2.90	0.01 ±0.00	0.01 ±0.00	0.01 ±0.00	0.01 ±0.00	0.29 ±0.19	0.30 ±0.18	5.46 ±1.32	5.46 ±1.31
	High	149.04 ±1.20	142.90 ±6.71	8.82 ±0.99	8.05 ±0.88	7.42 ±0.92	7.88 ±0.81	595.61 ±38.86	595.26 ±51.48	39.80 ±2.10	39.40 ±2.20	0.01 ±0.00	0.01 ±0.00	0.01 ±0.00	0.01 ±0.00	0.30 ±0.06	0.32 ±0.23	5.04 ±1.36	5.47 ±1.32

Note: The data were compared for statistical significance with the same-sex control group. In the same row, values with different small letter superscripts mean significant difference ($P<0.05$), while with the same or no letter superscripts mean no significant difference ($P>0.05$). The same as below. CON group, basal diet; Low group, basal diet + 2000 mg/kg; Middle group, basal diet + 10000 mg/kg. High group, basal diet + 50000 mg/kg. HGB =hemoglobin; RBC =red blood cell count; WBC =white blood cell count; PLT= platelet count; HCT= hematocrit; EOS= eosinophils; BAS =basophils; MO =monocytes (MO); and LY=lymphocytes.

Table S9. Effect of sublancin on the biochemical parameters of SD rats fed for 45 and 90 days during subchronic toxicity trial.

Items / Groups		ALB (mmol/L)		ALT (U/L)		AST (U/L)		BUN (mmol/L)		TC (mmol/L)		Cr (μmoL/L)		Glu (mmol/L)		TP（g/L）	
		♀	♂	♀	♂	♀	♂	♀	♂	♀	♂	♀	♂	♀	♂	♀	♂
		5	5	5	5	5	5	5	5	5	5	5	5	5	5	5	5
45 day	CON	28.54 ±1.80	27.75 ±6.66	33.71 ±3.14	34.15 ±4.04	124.01 ±10.30	116.47 ±14.11	5.93 ±1.29	4.86 ±0.34	1.59 ±0.28	1.69 ±0.24	46.59 ±4.88	47.80 ±4.06	4.01 ±0.55	4.86 ±0.34	45.67 ±1.81	44.91 ±6.62
	Low	29.57 ±2.61	30.94 ±4.90	33.65 ±2.31	34.41 ±1.38	123.09 ±9.00	113.15 ±14.28	5.36 ±0.96	4.63 ±0.60	1.74 ±0.20	1.60 ±0.28	48.99 ±3.43	46.41 ±3.83	3.76 ±0.65	4.74 ±0.50	45.77 ±2.62	48.12 ±4.86
	Middle	29.91 ±3.74	29.97 ±4.40	34.88 ±2.84	35.28 ±3.15	123.07 ±17.84	116.53 ±16.97	5.44 ±0.61	4.78 ±0.55	1.74 ±0.24	1.86 ±0.30	46.40 ±3.93	45.44 ±5.35	3.93 ±0.59	4.67 ±0.27	47.15 ±3.74	47.18 ±4.39
	High	29.49 ±3.05	28.88 ±2.71	31.41 ±1.74	35.23 ±3.64	120.19 ±15.50	109.74 ±9.74	5.26 ±0.34	4.42 ±0.40	1.66 ±0.36	1.60 ±0.42	45.60 ±3.70	44.12 ±2.54	4.49 ±0.52	4.47 ±0.74	47.19 ±2.54	44.12 ±4.77
90 day	CON	30.17 ±2.45	31.24 ±1.95	34.77 ±2.99	35.22 ±4.13	109.93 ±12.49	109.03 ±14.13	5.44 ±0.86	5.36 ±0.59	1.75 ±0.33	1.73 ±0.31	44.90 ±1.88	45.51 ±2.51	4.50 ±0.22	4.45 ±0.63	47.34 ±2.48	48.47 ±1.94
	Low	30.38 ±6.47	29.74 ±3.07	37.53 ±7.18	36.94 ±3.73	109.87 ±14.18	108.24 ±24.51	4.98 ±0.99	5.41 ±1.15	1.58 ±0.26	1.61 ±0.15	47.52 ±2.08	45.94 ±3.27	4.48 ±0.41	4.32 ±0.43	47.53 ±6.49	46.92 ±3.09
	Middle	32.29 ±4.86	29.20 ±2.65	38.38 ±5.20	36.77 ±4.50	105.66 ±12.77	108.32 ±14.45	4.70 ±0.47	4.71 ±2.72	1.55 ±0.11	1.62 ±0.21	45.02 ±3.48	45.67 ±3.04	4.44 ±0.57	4.71 ±0.95	49.40 ±4.94	46.38 ±2.64
	High	31.13 ±3.41	31.84 ±4.39	38.36 ±4.50	34.48 ±4.54	118.69 ±16.77	111.47 ±13.84	5.31 ±0.80	4.71 ±0.48	1.63 ±0.16	1.55 ±0.27	46.16 ±4.17	46.96 ±1.30	4.62 ±0.34	4.75 ±0.86	48.27 ±3.43	45.77 ±3.73

Note: The data were compared for statistical significance with the same-sex control group. In the same row, values with different small letter superscripts mean significant difference ($P<0.05$), while with the same or no letter superscripts mean no significant difference ($P>0.05$). The same as below. CON group, basal diet; Low group, basal diet + 2000 mg/kg; Middle group, basal diet + 10000 mg/kg. High group, basal diet + 50000 mg/kg. TP=total protein; ALB=albumin; ALT=alanine aminotransferase; AST= aspartate aminotransferase; BUN=blood urea nitrogen; TC= total cholesterol; Cr=creatinine; Glu=glucose; TG=triglycerides.

Table S10. Effect of sublancin on the serum biochemical parameters of SD rats fed for 60, 120 and 180 days during chronic toxicity trial

Groups	Items	ALB (mmol/L) ♀ 5	ALB (mmol/L) ♂ 5	ALT (U/L) ♀ 5	ALT (U/L) ♂ 5	AST (U/L) ♀ 5	AST (U/L) ♂ 5	BUN (mmol/L) ♀ 5	BUN (mmol/L) ♂ 5	TC (mmol/L) ♀ 5	TC (mmol/L) ♂ 5	Cr (μmoL/L) ♀ 5	Cr (μmoL/L) ♂ 5	Glu (mmol/L) ♀ 5	Glu (mmol/L) ♂ 5	TP (g/L) ♀ 5	TP (g/L) ♂ 5
60 day	CON	28.46 ±6.48	31.28 ±5.06	38.54 ±9.83	40.86 ±5.86	182.64 ±15.38	174.30 ±23.50	6.04 ±1.17	5.04 ±0.83	1.05 ±0.10	1.11 ±0.09	78.77 ±6.19	73.86 ±7.28	7.24 ±1.59	7.19 ±0.89	78.90 ±9.90	76.50 ±10.30
	Low	31.65 ±2.87	30.40 ±2.08	42.74 ±4.57	42.28 ±7.80	178.97 ±25.41	181.69 ±24.28	5.72 ±1.17	5.78 ±0.97	1.09 ±0.03	1.15 ±0.14	82.25 ±3.58	78.87 ±4.00	7.50 ±1.02	7.10 ±1.39	77.70 ±4.30	73.50 ±5.50
	Middle	32.84 ±4.12	32.52 ±5.30	40.70 ±5.38	40.10 ±7.34	175.61 ±16.58	177.67 ±17.21	5.24 ±0.94	5.90 ±0.81	1.10 ±0.03	1.07 ±0.06	82.52 ±8.22	80.17 ±6.20	7.71 ±0.82	7.28 ±1.80	79.30 ±5.70	77.80 ±5.40
	High	30.41 ±4.36	30.02 ±3.84	37.46 ±4.89	40.88 ±2.89	183.32 ±15.66	174.09 ±24.13	5.91 ±1.14	5.12 ±0.56	1.14 ±0.20	1.05 ±0.07	81.83 ±9.86	76.01 ±7.44	7.46 ±0.82	7.73 ±1.08	75.30 ±8.50	77.40 ±7.00
120 day	CON	33.62 ±2.88	34.04 ±4.66	38.54 ±9.83	40.50 ±10.79	170.81 ±17.72	179.36± 15.48	5.22 ±1.25	5.86 ±1.19	1.11 ±0.05	1.23 ±0.19	83.16 ±8.04	78.15 ±5.50	7.75 ±0.61	8.18 ±1.18	80.70 ±6.90	78.20 ±4.20
	Low	30.62 ±1.96	31.30 ±4.48	42.74 ±4.57	42.28 ±7.80	179.18 ±17.06	175.00 ±21.58	5.42 ±0.55	5.32 ±1.16	1.07 ±0.03	1.14 ±0.10	79.21 ±5.54	77.01 ±9.29	8.00 ±0.56	7.83 ±0.84	73.50 ±10.10	80.80 ±5.40
	Middle	32.94 ±4.77	31.78 ±2.62	41.52 ±6.30	43.50 ±7.72	178.88 ±23.17	180.26± 16.81	5.36 ±0.88	5.46 ±0.43	1.12 ±0.08	1.11 ±0.13	79.27 ±2.41	82.39 ±5.83	8.10 ±0.63	8.30 ±0.56	75.20 ±4.30	77.50 ±5.30
	High	32.87 ±3.53	33.45 ±5.83	41.70 ±5.38	40.10 ±7.34	175.61 ±22.91	176.78± 19.00	4.91 ±0.90	5.36 ±0.79	1.18 ±0.23	1.12 ±0.08	81.64 ±9.43	79.27 ±2.41	7.61 ±0.77	8.46 ±0.83	80.81 ±5.90	75.20 ±4.30
180 day	CON	34.90 ±2.40	34.42 ±4.44	43.80 ±8.60	41.30 ±7.10	179.29 ±23.01	170.46± 11.79	5.23 ±1.22	5.12 ±1.34	1.13 ±0.09	1.26 ±0.35	81.07 ±4.46	80.41 ±3.73	7.55 ±1.06	7.16 ±0.81	78.64 ±2.82	73.58 ±5.30
	Low	35.64 ±3.05	33.46 ±5.70	38.90 ±6.80	37.70 ±3.00	171.95 ±22.04	179.50 ±17.81	5.05 ±0.92	5.82 ±1.19	1.14 ±0.20	1.20 ±0.15	82.07 ±7.58	77.97 ±1.82	7.67 ±0.75	7.83 ±0.37	80.22 ±4.23	77.22 ±4.16
	Middle	32.71 ±3.80	31.36 ±4.50	39.70 ±7.60	41.30 ±7.50	182.41 ±16.00	175.45± 22.39	5.92 ±0.95	5.31 ±0.46	1.11 ±0.14	1.09 ±0.13	77.88 ±5.10	74.13 ±6.51	7.69 ±0.34	7.59 ±1.10	78.70 ±7.10	78.22 ±6.20
	High	34.02 ±2.65	32.40 ±4.07	42.10 ±7.90	39.70 ±3.80	181.01 ±24.04	172.74 ±10.53	5.07 ±0.94	5.65 ±1.42	1.16 ±0.18	1.27 ±0.30	78.17 ±4.87	78.64 ±3.11	7.98 ±0.67	7.26 ±0.89	79.90 ±3.16	73.52 ±5.26

Note: The data were compared for statistical significance with the same-sex control group. In the same row, values with different small letter superscripts mean significant difference (P<0.05), while with the same or no letter superscripts mean no significant difference (P>0.05). The same as below. CON group, basal diet; Low group, basal diet + 2000 mg/kg; Middle group, basal diet + 10000 mg/kg. High group, basal diet + 50000 mg/kg. TP=total protein; ALB=albumin; ALT=alanine aminotransferase; AST= aspartate aminotransferase; BUN=blood urea nitrogen; TC= total cholesterol; Cr=creatinine; Glu=glucose; TG=triglycerides.

Table S11. Effect of sublancin on the organ index of SD rats fed for 45 and 90 days during chronic toxicity trial.

Groups \ Items		Liver		Kidney		Spleen		Gut		Lung		Heart		Testicle	Ovary
		♀ 5	♂ 5	♀ 5	♂ 5	♀ 5	♂ 5	♀ 5	♂ 5	♀ 5	♂ 5	♀ 5	♂ 5		
45 day	CON	4.10±0.51	3.44±0.23	0.77±0.09	0.73±0.06	0.22±0.03	0.18±0.02	10.47±1.20	8.83±0.74	0.70±0.08	0.55±0.05	0.38±0.04	0.32±0.03	0.84±0.05	0.05±0.01
	Low	4.26±0.68	3.32±0.47	0.76±0.05	0.71±0.06	0.23±0.04	0.17±0.03	10.11±0.94	8.56±1.09	0.74±0.04	0.52±0.04	0.39±0.04	0.33±0.04	0.81±0.09	0.05±0.01
	Middle	4.01±0.30	3.47±0.41	0.76±0.07	0.68±0.12	0.21±0.04	0.18±0.03	9.78±1.10	8.55±0.64	0.71±0.09	0.52±0.03	0.38±0.04	0.31±0.04	0.83±0.10	0.05±0.01
	High	4.13±0.52	3.29±0.16	0.82±0.11	0.69±0.08	0.23±0.04	0.17±0.03	10.27±0.42	8.44±0.58	0.70±0.05	0.51±0.04	0.37±0.03	0.31±0.03	0.84±0.07	0.04±0.01
90 day	CON	4.58±0.40	3.19±0.25	0.75±0.08	0.65±0.06	0.22±0.03	0.14±0.03	11.59±0.97	7.95±0.81	0.85±0.04	0.55±0.05	0.46±0.05	0.30±0.01	0.64±0.06	0.05±0.01
	Low	4.46±0.58	3.15±0.15	0.74±0.05	0.68±0.05	0.20±0.03	0.13±0.02	11.14±1.33	8.12±0.46	0.85±0.06	0.56±0.01	0.45±0.04	0.31±0.03	0.64±0.04	0.05±0.01
	Middle	4.25±0.59	3.03±0.26	0.73±0.06	0.64±0.03	0.20±0.02	0.13±0.01	10.80±0.76	7.96±0.47	0.80±0.06	0.54±0.03	0.44±0.04	0.29±0.04	0.62±0.04	0.04±0.01
	High	4.28±0.50	3.02±0.15	0.77±0.03	0.69±0.08	0.20±0.03	0.14±0.03	11.20±1.30	7.80±0.42	0.79±0.09	0.53±0.04	0.43±0.04	0.30±0.02	0.67±0.07	0.04±0.01

Note: The data were compared for statistical significance with the same-sex control group. In the same row, values with different small letter superscripts mean significant difference ($P<0.05$), while with the same or no letter superscripts mean no significant difference ($P>0.05$). The same as below. CON group, basal diet; Low group, basal diet + 2000 mg/kg; Middle group, basal diet + 10000 mg/kg. High group, basal diet + 50000 mg/kg.

Table S12. Effect of sublancin on the organ index of SD rats fed for 60, 120 and 180 days during subchronic toxicity trial.

Groups	Items	Liver		Kidney		Spleen		Gut		Lung		Heart		Brain		Testicle	Epididymis	Ovary	Uterus
		♀ 5	♂ 5	♀ 5	♂ 5	♀ 5	♂ 5	♀ 5	♂ 5	♀ 5	♂ 5	♀ 5	♂ 5	♀ 5	♂ 5				
60 day	CON	4.39 ±0.39	4.24 ±0.34	0.85 ±0.12	0.81 ±0.05	0.23 ±0.02	0.19 ±0.03	10.06 ±1.44	8.51 ±0.49	0.91 ±0.13	0.71 ±0.09	0.34 ±0.03	0.31 ±0.03	0.54 ±0.08	0.34 ±0.03	0.86± 0.08	0.31± 0.05	0.05± 0.01	0.29± 0.03
	Low	4.37 ±0.41	4.29 ±0.47	0.85 ±0.09	0.82 ±0.08	0.23 ±0.03	0.21 ±0.03	10.29 ±1.11	8.93 ±1.62	0.88 ±0.14	0.77 ±0.04	0.37 ±0.05	0.33 ±0.05	0.57 ±0.05	0.35 ±0.05	0.82± 0.10	0.32± 0.05	0.04± 0.01	0.27± 0.04
	Middle	4.42 ±0.35	4.36 ±0.52	0.79 ±0.07	0.78 ±0.07	0.23 ±0.01	0.19 ±0.02	10.68 ±1.36	7.80 ±1.48	0.87 ±0.12	0.70 ±0.05	0.37 ±0.03	0.34 ±0.05	0.58 ±0.06	0.34 ±0.05	0.85± 0.10	0.31± 0.03	0.05± 0.01	0.29± 0.01
	High	4.25 ±0.23	4.19 ±0.31	0.84 ±0.12	0.81 ±0.05	0.24 ±0.04	0.19 ±0.01	10.09 ±1.08	8.84 ±0.77	0.96 ±0.05	0.67 ±0.06	0.36 ±0.05	0.29 ±0.02	0.58 ±0.08	0.33 ±0.05	0.81± 0.07	0.30± 0.04	0.05± 0.01	0.25± 0.04
120 day	CON	3.52 ±0.39	3.04 ±0.26	0.63 ±0.06	0.72 ±0.06	0.19 ±0.01	0.14 ±0.02	8.51 ±0.91	7.43 ±0.70	0.67 ±0.06	0.52 ±0.06	0.37 ±0.02	0.28 ±0.01	0.46 ±0.05	0.26 ±0.02	0.69± 0.04	0.28± 0.02	0.04± 0.01	0.20± 0.03
	Low	3.81 ±0.48	3.23 ±0.30	0.62 ±0.12	0.70 ±0.04	0.20 ±0.02	0.15 ±0.02	9.30 ±1.07	7.19 ±0.48	0.70 ±0.06	0.57 ±0.05	0.39 ±0.03	0.31 ±0.05	0.50 ±0.04	0.25 ±0.02	0.67± 0.04	0.29± 0.01	0.04± 0.01	0.22± 0.04
	Middle	3.83 ±0.26	3.19 ±0.39	0.67 ±0.06	0.68 ±0.03	0.20 ±0.02	0.15 ±0.01	9.29 ±1.42	6.78 ±0.50	0.72 ±0.06	0.52 ±0.07	0.39 ±0.02	0.29 ±0.04	0.47 ±0.07	0.24 ±0.03	0.67± 0.05	0.28± 0.03	0.04± 0.01	0.22± 0.03
	High	3.60 ±0.54	2.81 ±0.32	0.67 ±0.20	0.68 ±0.05	0.22 ±0.03	0.14 ±0.02	9.06 ±1.25	6.93 ±0.54	0.69 ±0.13	0.55 ±0.08	0.38 ±0.05	0.26 ±0.02	0.44 ±0.04	0.24 ±0.02	0.63± 0.08	0.26± 0.04	0.04± 0.00	0.21± 0.05
180 day	CON	3.70 ±0.34	2.78 ±0.27	0.67 ±0.11	0.59 ±0.07	0.19 ±0.03	0.12 ±0.01	9.61 ±0.82	6.10 ±0.30	0.84 ±0.07	0.51 ±0.04	0.41 ±0.03	0.27 ±0.01	0.40 ±0.02	0.24 ±0.03	0.61± 0.01	0.22± 0.02	0.04± 0.01	0.23± 0.03
	Low	3.62 ±0.38	2.83 ±0.16	0.67 ±0.09	0.60 ±0.04	0.19 ±0.06	0.12 ±0.01	9.78 ±1.53	5.99 ±0.27	0.79 ±0.07	0.49 ±0.05	0.41 ±0.03	0.31 ±0.04	0.41 ±0.07	0.27 ±0.02	0.63± 0.04	0.24± 0.02	0.04± 0.00	0.21± 0.04
	Middle	3.60 ±0.24	3.01 ±0.41	0.61 ±0.04	0.60 ±0.03	0.18 ±0.03	0.12 ±0.01	8.95 ±0.67	6.07 ±0.48	0.79 ±0.07	0.49 ±0.04	0.41 ±0.03	0.27 ±0.02	0.42 ±0.05	0.26 ±0.04	0.60± 0.06	0.22± 0.01	0.04± 0.00	0.21± 0.03
	High	3.80 ±0.36	3.04 ±0.41	0.66 ±0.07	0.63 0.06	0.19 ±0.02	0.13 ±0.01	9.74 ±1.45	6.43 ±0.62	0.80 ±0.08	0.48 ±0.10	0.38 ±0.06	0.27 ±0.05	0.43 ±0.08	0.22 ±0.03	0.60± 0.04	0.24± 0.04	0.04± 0.01	0.20± 0.03

Note: The data were compared for statistical significance with the same-sex control group. In the same row, values with different small letter superscripts mean significant difference ($P<0.05$), while with the same or no letter superscripts mean no significant difference ($P>0.05$). The same as below. CON group, basal diet; Low group, basal diet + 2000 mg/kg; Middle group, basal diet + 10000 mg/kg. High group, basal diet + 50000 mg/kg.

Disclaimer/Publisher's Note: The statements, opinions and data contained in all publications are solely those of the individual author(s) and contributor(s) and not of MDPI and/or the editor(s). MDPI and/or the editor(s) disclaim responsibility for any injury to people or property resulting from any ideas, methods, instructions or products referred to in the content.
